# Decentralization and efficiency of subsidy targeting: Evidence from chiefs in rural Malawi^☆^

**DOI:** 10.1016/j.jpubeco.2019.07.006

**Published:** 2020-05

**Authors:** Maria Pia Basurto, Pascaline Dupas, Jonathan Robinson

**Affiliations:** a Universidad del Pacífico, Lima, Perú; b Stanford University, United States of America; c NBER, United States of America; d University of California, Santa Cruz, United States of America

**Keywords:** Nepotism, Productive efficiency, Political economy, Agricultural inputs, Chiefs, Subsidies

## Abstract

Lower-income countries spend vast sums on subsidies. Beneficiaries are typically selected via either a proxy-means test (PMT) or through a decentralized identification process led by local leaders. A decentralized allocation may offer informational advantages, but may be prone to elite capture. We study this trade-off in the context of two large-scale subsidy programs in Malawi (for agricultural inputs and food) decentralized to traditional leaders (“chiefs”) who are asked to target the needy. Using household panel data, we find that nepotism exists but has only limited mistargeting consequences. Importantly, we find that chiefs target households with higher returns to farm inputs, generating an allocation that is more productively efficient than what could be achieved through strict poverty-targeting. This could be welfare improving, since within-village redistribution is common. Productive efficiency targeting is concentrated in villages with above-median levels of redistribution.

## Highlights

• Chiefs do not do as well as a PMT when asked to target subsidies to the poor.
• Chiefs favor their kin. Nepotism does not contribute much to poverty mistargeting since chiefs' kin are as poor as other villagers.
• Chiefs do not target only on poverty; they target input subsidies to people with higher returns to agricultural inputs.
• Such productive efficiency targeting is observed primarily in villages that exhibit above-median levels of income-pooling.

## 1 Introduction

Targeting programs such as subsidies to needy households is an important part of what governments do. To do this effectively, governments must first identify who is truly needy, which is difficult in developing countries where government infrastructure and information technology are limited (particularly in rural areas). Governments typically have the choice to administer such selection of eligibles centrally, or to decentralize authority to local communities (usually these programs are officially administered by local leaders).^1^ Decentralization has two main benefits: (1) local leaders are likely more informed about the relative neediness of people in their village (especially in a context in which most people do not file a tax return); and (2) local leaders will be more accountable to villagers, particularly if leaders face village electoral pressure or are motivated by reputation concerns. On the downside, decentralization may open the door for corruption or nepotism.

This paper uses rich panel data collected from a sample of 1559 households over four survey rounds in 2011–2013 to explore this fundamental trade-off in the context of two subsidy programs in Malawi — the well-known farming input subsidy program (FISP) which provides subsidies for fertilizer and hybrid seeds once a year, and a one-time food aid relief program put in place after a financial crisis and drought in 2012. These programs were conceived as anti-poverty programs and the selection of beneficiaries was decentralized to local traditional leaders, called chiefs. How well do chiefs target the programs?

This is a setting in which the trade-off between nepotism and information could be severe. On the one hand, nepotism is possible since chiefs cannot be held accountable via electoral pressure — in contrast to the contexts studied in Bardhan and Mookherjee, 2000, Bardhan and Mookherjee, 2005 or Bardhan (2002), the position of chief in Malawi, as in many other countries in the region, is hereditary and chiefs face fairly weak oversight. There is also no strict eligibility rule provided by the government (only general guidance on who should be “considered” for the subsidy) and no government back-checking of allocations.^2^ But on the other hand, local information is critical, along two main dimensions: (1) shocks occur frequently and chiefs likely have good information on recent household-specific economic conditions; and (2) the return to inputs will likely be heterogeneous across households within a village and related to factors such as household demographics (especially in regards to availability of family labor), soil type, and access to credit. Targeting inputs to those with the highest returns will increase total village output by the most, and if *ex post* inter-household transfers can be used to redistribute these gains, then targeting based on productive efficiency rather than neediness may be Pareto-optimal.

The paper answers two sets of questions. First, how common are errors of exclusion (truly needy households not getting the subsidy) under the status quo? Do chiefs use local information to target households which have suffered recent negative shocks? Do they favor relatives? Second, do chiefs take into consideration productive efficiency when allocating the input subsidies? Specifically, do they target the agricultural subsidies to households with higher returns to fertilizer?

To answer the first set of questions, we use observed food expenditures in the immediate pre-subsidy period as our measure of neediness, and benchmark the targeting effectiveness of the chiefs against that of a counterfactual proxy-means test (PMT). We find evidence that both the chiefs and the counterfactual PMT miss a substantial fraction of poor people, but that the chiefs miss significantly more: chiefs make more and bigger errors. Specifically, mean-squared error is 2–3.5 times higher for the observed allocation as for a counterfactual PMT. We also find evidence of nepotism: chiefs are more likely to target food subsidies to relatives. However, this nepotism appears to have minimal aggregate welfare consequences, since chiefs' relatives are similarly poor as other villagers. We also find that chiefs use their informational advantage to the benefit of households hit with negative shocks: people who have experienced droughts, floods, cattle death, or crop disease are significantly more likely to receive subsidies under the chief than under a PMT-based allocation.

The second part of the paper tests whether chiefs target input subsidies to people with higher returns to agricultural inputs. The test is derived from a model of subsidy allocation in which chiefs have preferences over households, but also have information about household-specific returns to agricultural inputs. We assume that there is little heterogeneity in productive returns to food, in which case the allocation of the food subsidy is reflective of the welfare weights. To back out the relative importance of productivity considerations in the chief's objective function, we exploit the wedge between the allocations of the food and input subsidies. Taking this to the data, we find that chiefs indeed allocate relatively more inputs to households with higher gains from fertilizer use, while the PMT would not, suggesting productive efficiency gains from a decentralized system.^3^ As predicted by the model, targeting based on gains to fertilizer use is observed primarily in villages that exhibit above-median levels of income-pooling: it is only if the extra production can be shared ex post through inter-household transfers that targeting agricultural subsidies based on efficiency rather than poverty considerations can be Pareto-optimal.

Our paper paints a nuanced view of the targeting of chiefs. On the one hand, we find evidence that decentralization has the benefit of improved information on recipients.^4^ On the other hand, we do find evidence of nepotism. As in Alatas et al. (2013), we find that the ultimate welfare consequences of nepotism are likely small, however, since a PMT would not perform much better.^5^ The main reason for this is that assets like land are noisy predictors of consumption in rural Africa — the R-squared for our PMT regression is only 0.32, and we document similar figures for datasets from Kenya and Uganda. This may be one reason why earlier work — including several previous studies in Malawi (Dorward et al., 2008, Dorward et al., 2013, Kilic et al., 2013) had found higher levels of mistargeting and elite capture than we do: they used *assets* as a proxy for need instead of consumption.

Our paper makes several contributions to the literature. Our core contribution is to bring attention to the difference between poverty-targeting and *poverty reduction* (the ultimate goal of subsidy programs). In communities with informal income-pooling, productive efficiency targeting may be the more effective (albeit indirect) way of reducing poverty. For this reason, looking only at who gets input subsidies rather than how the produced output is allocated is not sufficient to gauge impacts on poverty alleviation.

More broadly, we contribute to the literature on the role of traditional authorities in African development. While survey evidence from the Afrobarometer suggests that traditional leaders are perceived to regulate important aspects of the local economy in numerous African countries (Logan, 2011, Michalopoulos and Papaioannou, 2013), the question of whether their existence further undermines weak governance, or instead palliates it, is still unsettled. Acemoglu et al. (2014) find that areas of Sierra Leone where competition among potential chieftaincy heirs was low during and after British colonial rule have significantly worse development outcomes today, but higher levels of respect for traditional authorities. They hypothesize that this reflects the ability of uncontested traditional ruling families to simultaneously capture resources and civil society organizations. Our evidence from Malawi mitigates this view: in our context, traditional leaders are uncontested and popular, as in Acemoglu et al. (2014), but effective at targeting input subsidies to productive farmers, possibly putting their village on a higher growth path.^6^

The layout of the paper is as follows. Section 2 presents some background on the Malawian local governance structure and decentralized subsidy programs. Section 3 discusses the sample and data. Section 4 presents evidence on poverty-based (mis)targeting. Section 5 tests for productive efficiency targeting. Section 6 concludes.

## 2 Institutional background

### 2.1 Local governance in Malawi and the role of chiefs

In Malawi, the democratically elected local government coexists with a traditional chieftaincy hierarchy. There are four ranks within this hierarchy: Paramount Chief, Traditional Authority (TA), Group Village Headman (GVH), and Village Headman (also known as village chief). In our data, TAs have authority over areas smaller than a district. They oversee from 10 to 45 GVHs, and GVHs oversee between 2 and 10 villages.^7^

Chiefs in Malawi hold little formal power. They do not have direct control over any public funds and are not allowed to raise local taxes. However, chiefs hold other customary responsibilities. The 1998 Decentralization Policy and Local Government Act Malawi Government (1998) recognized the rights of chiefs to allocate communal land and adjudicate matters related to customary law (in particular customary land). Chiefs also play an advisory and coordination role regarding local development projects. Finally — and this is the focus of our paper — chiefs are typically relied upon to identify beneficiaries for targeted government programs.

Traditional leadership positions are hereditary, and chiefs who pass away are replaced from within the chieftaincy clan.

Chiefs are paid a salary by the government that is known as *mswahala*, but it is fairly small.^8^ Chiefs do occasionally charge fees to villagers (in our sample, 44% of villagers report having ever made a payment to the village chief). Interestingly, chiefs are favorably viewed by the majority of the Malawian population. In 2008–2009, 74% of Afrobarometer respondents in Malawi perceived traditional leaders as having “some” or “a great deal” of influence, and 71% thought the amount of influence traditional leaders have in governing the local community should increase — for comparison, the average across 19 African countries for these two questions were both 50% (Logan, 2011). Possibly as a result of this high popularity, chiefs appear able to influence local villagers on whom to support in general elections and local government elections (Patel et al., 2007), an influence that may limit their accountability to elected representatives.

### 2.2 Subsidy programs

#### 2.2.1 Fertilizer subsidy program

Malawi's Farming and Agricultural Input Subsidy Program (FISP) is one of the largest fertilizer and seed subsidy programs in the world.^9^ Though the program has existed since 1998, it greatly expanded after a drought in 2004 and steadily increased in size for a number of years after, until contracting more recently. In 2012–2013, the program reached 4.4 million recipients and took up 10–15% of the government's budget (Dorward et al., 2013, Baltzer and Hansen, 2011). In our data, the percentage of people benefiting from subsidies has increased steadily over time, from 63% in 2008 to 82% in 2012.

The subsidy program covers several inputs and comes in the form of vouchers, which are redeemable at local agricultural shops. The four items covered by the voucher subsidy during our study period were planting fertilizer (a 50-kilogram bag of NPK, worth about $40 at market prices in 2013), top-dressing fertilizer (a 50-kilogram bag of Urea, comparable in price to NPK), hybrid maize seeds (a 5-kilogram bag, worth about $7), and hybrid groundnut seeds (a 2-kilogram bag, worth $2.60). The price of the voucher is only $1.7, so the subsidy was worth about 98% of the value of the input during this time period. As a result, take-up of the vouchers in our study sample is universal.^10^

There is no strictly defined, official eligibility criteria for the subsidy, but the intention is to target the poor and vulnerable. The official FISP guidelines reads that beneficiaries “will be full time resource poor smallholders Malawian farmers” but no threshold is provided for what defines “resource poor.” The program guidelines does hint at particular groups however: “...the following vulnerable groups should also be considered: elderly, HIV positive, female headed households, child headed households, orphan headed households, physically challenged headed households and heads looking after the elderly and physically challenged” (MoAFS, 2009). Many of these targeted groups may have lower returns to inputs than the average poor household, for example because they are unable to farm intensively.

The identification of beneficiaries has three main stages (Chirwa et al., 2010). First, the government conducts a national farmer registration census. Second, the central government allocates vouchers to districts as a function of the area's farming population and the acreage under cultivation.^11^ Finally, within each village, once the number of subsidies available to the village is known, a list of eligible villagers is made. Formally, the selection of beneficiaries at this stage is supposed to be done by a Village Development Committee through open community meetings, and audited by the DADO. However, as we will show below, most authority appears to be *de facto* delegated to chiefs.^12^ Once the list of beneficiaries have been received by the DADO, it establishes a date and venue for the distribution of the vouchers themselves. The distribution is done by a staff member from the DADO. Listed beneficiaries have to show their voter registration card in order to receive the vouchers and also to redeem the vouchers at the retail stores (MoAFS, 2009).

The identification of beneficiaries and distribution of vouchers is timed to precede the main rainy season (which runs from planting in November/December until harvest in April–August). During our study period, subsidy vouchers were distributed in September/October, in advance of planting.

#### 2.2.2 Food subsidy program

Malawi devalued its currency in 2012, causing prices to rise 20–30% in 2012–2013 (World Bank, 2015), which made food imports prohibitively costly. There was also a poor harvest in 2012, caused by a drought. In response, a food subsidy program was implemented in late 2012, lasting from November 2012 to January 2013. In our area of study, the subsidies were distributed in kind. As with the input subsidy, the program was targeted at the “poor” but without a precise threshold or formula for what constitutes poverty. Of those receiving the subsidy in our data, the average amount received was 103 kg of maize, 14 kg of soy blend, 18 kg of pigeon peas, 10 kg of beans, and 3 L of oil. We estimate that this package was worth about $72 in 2013 USD. As with the farming input subsidy program, chiefs were given primary responsibility for identifying which households would receive the food aid.

## 3 Data

### 3.1 Sample

The data we use for this paper was collected as part of a separate randomized controlled trial to estimate the impact of providing savings accounts to unbanked households (Dupas et al., 2018, henceforth DKRU). The project took place around the catchment areas of NBS bank branches in two districts of Southern Malawi — Machinga and Balaka. The sampling frame for DKRU relied on a census of market businesses and a census of households conducted at the end of 2010 — we use only the household sample for this analysis. The household census listed 9297 households from 68 villages in three Traditional Authorities (TA) areas: Kalembo, Sitola, and Nsamala. Of these, 78.8% met the eligibility criteria set by DKRU: they did not have a bank account and had a female head of household. DKRU randomly selected a subset of this sample for project inclusion, and completed baseline surveys with 2107 households. This set of households is uses for the analysis in this paper, though we must drop some households because their data is incomplete.^13^ We are ultimately left with 1559 households in 61 villages for our analysis.

Given this sampling frame, our data departs from the universe of villagers in two ways. First, we systematically excluded villagers who had bank accounts at baseline (which was about 15% of the sample). These individuals are certainly richer than the average villager, and for this reason our analysis may underestimate targeting errors (if any of the people with bank accounts ended up receiving subsidies).^14^ Second, even among unbanked households, our dataset includes only a subset of people in each village (roughly 10% on average). However, since these villagers are randomly selected, our results are still internally valid and of interest — our goal is to understand how chiefs allocated subsidies *within this sample*, and our basic thought experiment is to ask what the gains would be from re-allocating subsidies *within this sample*.

### 3.2 Data sources

#### 3.2.1 Household panel

We have four waves of survey data for each household: (1) a baseline conducted from February to March 2011; (2) a first follow-up survey conducted from February to March 2012; (3) a second follow-up survey conducted from September to December 2012; and (4) an endline survey conducted from February to May 2013. The baseline survey includes a standard set of demographic variables, including a module on asset ownership which can be used to construct the allocation that would have obtained under a counterfactual allocation based on a proxy-means test from baseline assets. Each of these survey rounds included detailed expenditure modules.

The follow-up and endline surveys include a module on the farming subsidy. This is used to construct a time series of subsidies received from 2008 to 2013, for each household. The module includes information on which input subsidy was received, whether the household received the voucher itself or shared another household's voucher, and what the household actually did with the subsidized products (used them, sold them, shared them, etc.). The endline survey also asked these questions for the food subsidy, which was introduced in 2012. Finally, the endline included a separate module with questions on how the input and food subsidies were allocated. These include questions on how (in the respondent's opinion) the vouchers were allocated, whether a public meeting was held, whether the respondent participated in the meeting, etc.

In addition, between August and October 2014 we collected a fifth wave of data for a random subset of 563 households in the initial sample. This survey asked additional questions on the process through which subsidies were allocated and on respondents' attitudes towards the allocation process as well as their perception of their chief's role, beliefs and objectives in this allocation. Importantly, we also elicited households' beliefs on the returns to farming inputs on their own land.

#### 3.2.2 Chiefs survey

Between August and October 2014 we collected surveys with all of the 105 traditional leaders in our study area of 61 villages, including 76 village headmen (chiefs) and 29 group village headmen (GVH).^15^ The survey included questions on their tenure and responsibilities, and included questions about the details of how the FISP and food subsidy programs were allocated. We also measured chiefs' self-reported awareness of whether some farmers had higher returns to inputs than others, and their knowledge of shocks encountered by villagers.

### 3.3 Characteristics of households, chiefs and villages

Table 1 presents basic summary statistics on the households in our sample. Panel A includes time-invariant characteristics collected at baseline. The first variable shown is the household's self-reported relationship to the chief. We asked the following question to each respondent: “Are you related to the chief?,” to which 27% reported yes. In a follow-up question, we asked: “How are you related?” The modal answer was the chief is an uncle (20% of the related cases), followed by brother (13%), brother-in-law (12%) and grandfather (12%). In what follows, we refer to those who reported as being related to the chief as “kin”.^16^

**Table 1:** Summary statistics on households in the sample.

	(1)	(2)	(3)	(4)	(5)
			Difference		
	Overall mean	Std. dev.	Kin vs. non-kin		Correlation between rounds
			Diff.	Std. err.	
*Panel A. Time-invariant baseline variables*					
Related to chief (“kin”)	0.27	–			
Mud/dirt floor	0.90	–	0.02	0.02	
Thatch roof	0.77	–	0.01	0.02	
Has electricity in dwelling	0.006	–	0.002	0.004	
Reads or writes chichewa	0.58	–	−0.07	0.029*	
Years of education	4.86	3.54	−0.50	0.205*	
Widowed or divorced female	0.29	–	0.03	0.03	
Household size	4.57	2.07	−0.06	0.12	
Number of children	2.49	1.72	−0.06	0.10	
Respondent age	40.14	17.09	0.50	0.99	
Owns land	1.00	–	0.00	0.00	
If yes, acres of land owned	2.36	1.97	0.18	0.11	
Value of durable assets owned (USD)	98.04	384.06	−11.27	22.32	
Value of animals owned (USD)	36.76	105.51	−2.43	6.15	

*Panel B. Time-varying variables*					
Total expenditures per capita (monthly)^a^	9.66	10.85	−0.476	0.313	0.45
Total food expenditures per capita (monthly eq.)	6.80	7.77	−0.349	0.224	0.35
PCF: Total non-staple food expenditures per capita (monthly eq.)	2.95	3.64	−0.216	0.105**	0.44
*Shocks*					
Experienced drought or flood (past 3 months)	0.28	–	0.005	0.013	−0.33
Experienced cattle death or crop disease (past 3 months)	0.20	–	0.013	0.012	0.04
Respondent missed work due to illness (past month)	0.26	–	−0.002	0.015	0.16
Other household member was sick (past month)	0.69	–	0.007	0.013	0.16
Report being worried about having enough food to eat (past month)	0.72	–	−0.023	0.012	0.14
Share of days with enough food to eat	0.67		0.004	0.016	0.19
*Informal redistribution*					
Received transfers from other villagers in past 90 days	0.58		−0.017	0.014	0.11
Made transfers to other villagers in past 90 days	0.25		−0.003	0.013	0.07
Number of observations	6236				
Number of households	1559				

*Panel C. Reported returns to fertilizer (2014 survey)*					
Self-reported total production without fertilizer use (50-kilogram bags)	3.87	2.62	0.25	0.25	
Self-reported total production with fertilizer use (50-kilogram bags)	18.48	9.41	0.42	0.87	
Gain in production from using fertilizer (50-kilogram bags)	14.50	8.05	0.20	0.76	
Gain in production from using fertilizer (50-kilogram bags), per acre	7.83	4.92	0.20	0.47	
Number of households	532				

Note: All monetary amounts are in US dollars. Years of education is highest in the household (husband or wife).

a Expenditures are winsorized at the 99th percentile.

Households in the sample are very poor: 90% have mud floors or worse quality, 77% have thatch roofs, and less than 1% have electricity. Only 59% are literate, and average years of education for the household head is just below 5.^17^ Twenty-eight percent of households have no male head (most of these households are likely widows), and 97% own land.

Panel B shows time varying expenditures, shocks and transfers. Across rounds, households report spending only $9.66 per month per capita in total, and the majority of this is on food ($6.80). These figures place these households well below the global extreme poverty threshold of $1.25 per day. Shocks are also quite common: 26% of respondents lost at least 1 day of work in the past month due to illness, 69% of respondents experienced the sickness of another household member in the past month, 28% experienced a drought or flood in the past 3 months, and 20% experienced crop loss or livestock death in the past 3 months. Across survey rounds, 72% of households report being worried about having enough food to eat in the past 3 months. Transfers across households within the village are very common, with 58% of households reporting being recipients of transfers in the last 90 days, and 25% reporting having made transfers.

Columns 3 and 4 of Table 1 show, for each variable, the gap between kin and non-kin and its standard error. This reveals that if anything, kin are poorer than non-kin — they are significantly less educated (Panel A), and have slightly lower consumption (Panel B). Lastly, Column 5 shows the correlation between survey rounds for the variables in Panel B. This shows quite a bit of variability over time — the inter-round correlation in food expenditures is only 0.35–0.43, suggesting that neediness varies over time.

Table A1 presents summary statistics on villages and village chiefs. The average village in our sample has 309 households and over 7000 acres of customary land. The average village chief is 53 years old and has about 5 years of education. Eighty-two percent of chiefs are male. The average chief has been in power for about 13 years, and 90% inherited the position (most of the remainder were appointed). The vast majority faced no competition from within the family blood line for the position. In principle, traditional leaders can be removed from office or reprimanded, but our data suggests this almost never happens: only one chief reported every being suspended. When chiefs were asked about their main responsibilities, the five most common responses were resolving conflicts among villagers (90%), reporting issues to higher level chiefs (61%), monitoring village projects (56%), disseminating information to villagers (33%), and overseeing subsidy programs (20%).^18^

### 3.4 Summary statistics on the allocation of subsidies in our sample

Table A2 presents summary statistics on the process through which input and food subsidies were allocated. Panels A and C rely on the latest round of survey data (2014) and presents evidence on how both chiefs (Panel A) and villagers (Panel C) experience and perceive the subsidy allocation mechanisms. Panel B presents data from the earlier household survey waves.

The data confirms that chiefs are the primary decision-makers in allocating subsidies. Turning first to Panel A, the majority of village chiefs report that they have control over the subsidy allocation: 62% declare that they decide by themselves, while an additional 3% report that they decide in collaboration with others. Of the remainder, 13% report that the village development committee (of which the chief is a member) decides the allocation, and 13% report that subsidies are allocated in a village meeting (which the chief typically runs). When asked about selection criteria, chiefs report need as the primary criterion. Chiefs also put significant weight on female-headed households, households which recently received a shock households taking care of orphans, and households that the chief believes are hard-working.

Panel B shows that community meetings regarding selection happen quite regularly: 95% of villagers report that a meeting was held, and 82% report attending this meeting for FISP (65% attended in regards to the food subsidy). Consistent with chief responses in Panel A, households responses in Panel C confirm that the chief is mostly responsible for allocating the subsidies — 72% report that the chief decides alone (49%) or with others (23%) on the input subsidies, and 73% report that the chief alone decides on the food subsidies. Households report similar inclusion criteria as do chiefs (needy households, as well as elderly and female-headed households).

While official FISP guidelines do not endorse sharing of subsidy packages, we find strong evidence that sharing is in practice very common (Web Appendix Table W1). Seventy-seven percent (0.46/0.60) of households who received an input subsidy voucher report sharing it. Moreover, we find that sharing is often at the direction of the chief: of those who shared, 83% say they received instructions from the chief on whether to share it, and 79% received specific instructions from the chief on *whom* to share with. Food subsidies are similarly shared.^19^

In what follows, we perform all analyses considering both allocations: the allocation of the vouchers themselves, and the allocation observed *after* sharing (we call this the “realized allocation”).^20^

## 4 Poverty-targeting

### 4.1 Measuring neediness

To measure neediness, we use food expenditures, which we consider a proxy for consumption. Food expenditures have been shown to be better predictors of neediness than other measures such as income (Deaton, 1997, Meyer and Sullivan, 2012).

While we measured expenditures on 12 broad food categories (covering all food types), in the main analysis we focus on the 10 categories that are typically purchased rather than self-produced.^21^ These 10 categories are vegetables, fruits, meat, dairy/eggs, salt, sugar, other cooking items (oil, margarine), coffee and tea, snacks, and juice/sodas.

We compute the sum of expenditures on these 10 food categories over the 30 days preceding the survey and then divide the sum by the number of household members to construct “per capita non-staple food expenditure” or PCF, our measure of need going forward (we report this figure in USD).^22^ The distribution of log PCF in our data is plotted separately for the two main years of analysis, 2011 and 2012, in the top panel of Web Appendix Fig. W.

#### 4.1.1 Timing

The food expenditure we would ideally use to determine “true need” (PCF eligibility) would be measured at the time that subsidy beneficiaries are identified (which is around August for the input subsidy and November for the food subsidy). The timing of our surveys does not precisely correspond to these periods. Our food expenditure module covered the last 7–30 days (depending on the question) before the survey date. Thus, given the dates of the surveys mentioned in Section 3.2, we have consumption data for the following periods: January 2011 to February 2011; January 2012 to February 2012; August 2012 to November 2012; and January 2013 to April 2013. To study the targeting of the 2011 input subsidy, we thus have to rely on the January 2011 to February 2011 expenditure data, which is substantially before the period of interest. In particular, it is before the March 2011 harvest, which is likely an important determinant of actual neediness as of August–November 2011.^23^ Fortunately, the data used for the 2012 subsidies is for the correct time period (August to November). For this reason, our 2012 results are our preferred estimates.

### 4.2 Constructing counterfactual allocations

#### 4.2.1 Neediness rank

For each village, we observe the total number of households within our sample who received a voucher — we call this number $\bar{s}$. To construct the counterfactual in which vouchers were distributed based on true consumption, we rank households (within each village) by their per capita non-staple food expenditure (PCF). We consider a household “PCF eligible” if they are ranked at or below the $\bar{s}$th farmer in the PCF distribution (breaking ties based first on total food expenditures and second on total expenditures on all items). We also repeat the same procedure for whether households actually received inputs or food (i.e. either by directly receiving a voucher or indirectly because a voucher recipient shared with them) to construct the counterfactual for the realized allocation.

#### 4.2.2 PMT score rank

To construct the counterfactual in which subsidies were allocated via PMT, we repeat this procedure but this time we rank households (within each village) by a “PMT score.” We compute the PMT score as follows: we regress log PCF on household characteristics, including demographic characteristics, dwelling characteristics, assets and occupation, and use the estimated coefficients to predict a score for each household. As in Alatas et al. (2012), we do this in two steps: we first run kitchen sink regressions with all available characteristics and then, using a backward step-wise procedure, keep only those characteristics which are statistically significant at the 10% level.

PMT regressions are shown in Table A3. We show the results for both per capita and per adult equivalent food expenditure, and find slightly higher predictive power for per capita values.^24^ From Column 1, we obtain a R-squared of 0.32, which is somewhat lower than the 0.40 obtained by Alatas et al. (2012) in Indonesia (when pooling districts together).^25^ For comparison, we also construct a PMT score using data from the 2010–2011 wave of the Integrated Household Survey (IHS3), a representative household survey collected by Malawi's National Statistics Office. We restrict that dataset to the two districts in our sample, and estimate PMT regressions using the same backward step-wise method to identify covariates. Results are shown in Web Appendix Table W2. In the table, we run regressions separately where we restrict to only those variables which were also collected in our surveys (which we call “BDR variables”), which are shown in Column 1, and for all potential covariates available in the IHS3 (Column 2). We find R-squared statistics in both regressions of approximately 0.4. We conjecture that the somewhat lower R-squared we observe in our own survey data is because our sample is somewhat poorer than a representative sample, and their consumption may be more volatile due to lower access to insurance. To shed some light on this, we run similar regressions in samples of unbanked households we have collected in other work in Kenya (Dupas et al., 2019) and Uganda (Dupas et al., 2018). We find an R-squared of 0.31 in Kenya and 0.28 in Uganda.

### 4.3 Poverty-targeting results

#### 4.3.1 PMT vs. chief allocation

**Fig. 1:**
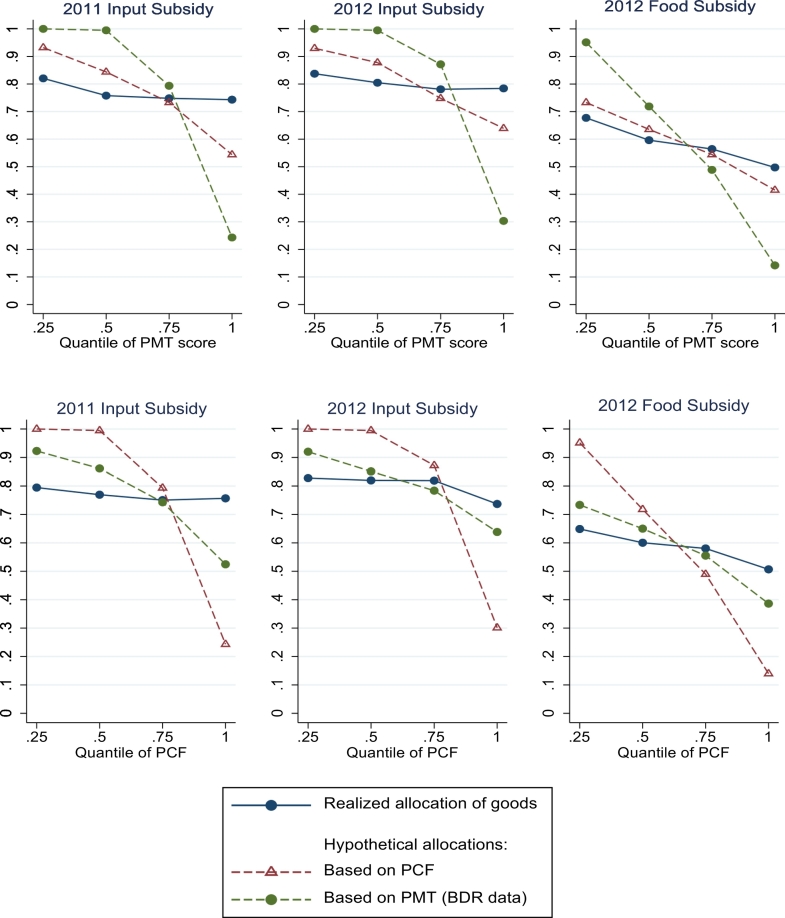
Comparing realized allocation to counterfactual allocations. Notes: See main text in Section 4.2. The PMT formula is obtained using 2011 data. The PCF is contemporaneous of the subsidy allocation decision. The chief allocation is the allocation observed, made by chiefs. Because the share of households that receive subsidies vary across villages, the threshold PMT (PCF) score for eligibility varies across villages, which explains why the allocations by PMT (PCF) quantile are not either 1 or 0.

Our first set of results is shown in Fig. 1, which plots the probability of receiving the subsidies by quintile of the PMT score distribution (top panel) and quintile of the PCF distribution (bottom panel). These quintiles are across the entire sample, and so include across-village variation. We show the realized allocation (i.e. the allocation after vouchers were shared) as well as two counterfactual allocations: the PMT allocation, our “benchmark” for what could be done under centralization; and the PCF-based allocation, the “optimal” allocation. We pool across villages, which vary in their underlying distributions as well as in the number of subsidies available, which explains why neither of the two counterfactual allocations are perfect step functions of their respective distributions. It also explains why even the PCF-based allocation in Fig. 1 does not reach perfect targeting: there is mistargeting of the number of subsidies across villages, which means that even a perfect allocation within village would yield evidence of mistargeting. The gradient in the PCF-based allocation in Fig. 1 should therefore be considered as the “best possible targeting” given the across-village allocation in our data.^26^

From the top panel of Fig. 1, it is clear that chiefs target different people than the PMT would: while the PMT, by definition, would allocate subsidies to 100% of people at the bottom of the distribution, the chiefs' allocation has a much flatter gradient with respect to the PMT score. In isolation, this result looks similar to Dorward et al., 2008, Dorward et al., 2013 and Kilic et al. (2013), who look at how well chiefs target based on assets and conclude that there is widespread mistargeting.

The bottom panel of Fig. 1, which show targeting based on PCF, also show that the PMT does better than chiefs — but the gap is much smaller than in the top panel. In the allocation decision of 2011 (which was contemporaneous to the survey from which the PMT was calculated), the gradient for the PMT allocation is quite a bit steeper than that of the chiefs, but by 2012 the slopes are more similar. This could be because characteristics measured in 2011 become less and less predictive as time goes on, and might suggest that the advantage of a PMT may be short-lived. Fig. 1 also shows that the PMT makes a substantial number of errors. This is true even if we use the PMT formula from the IHS3 rather than the one derived in our dataset. The relatively poor targeting performance of the PMT seems due to the fact that assets (the most important factor in the PMT) are a relatively poor proxy for need in our study context, because PCF eligibility is not time-invariant (the correlation between food expenditures across rounds is only 0.35 as previously discussed and shown in Table 1) and because there are important unobservables in the determinants of PCF.

**Fig. 2:**
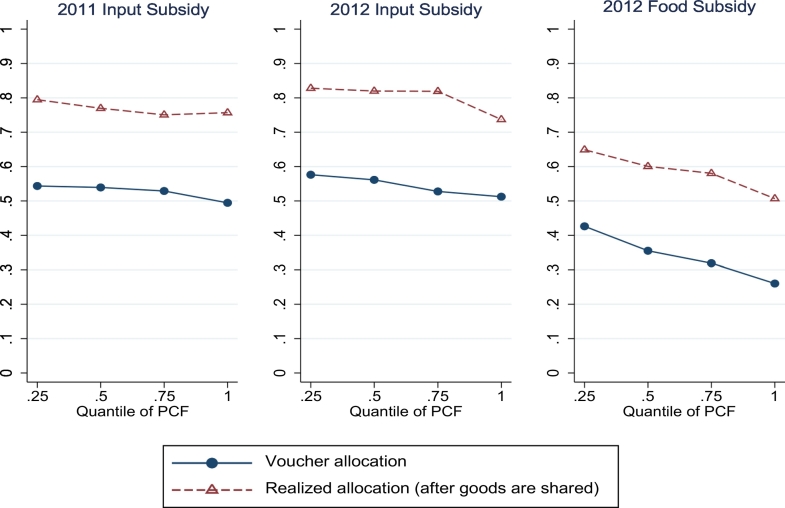
Allocation of vouchers vs. realized allocation of goods. Notes: See main text in Section 4.2. The PMT formula is obtained using 2011 data. The PCF is contemporaneous of the subsidy allocation decision. The chief allocation is the allocation observed, made by chiefs. Because the share of households that receive subsidies vary across villages, the threshold PMT (PCF) score for eligibility varies across villages, which explains why the allocations by PMT (PCF) quantile are not either 1 or 0. High sharing villages are those where the number of transfers to other households, as a fraction of village size, is above the village-level median.

In Fig. 2 we show the allocation against the PCF quantile, both *before* and *after* sharing. Before sharing, just over 50% of households received the input voucher and 34% received the food voucher; after sharing, these percentages increase to about 78% and 59%. However, poverty-targeting efficiency does not improve from sharing: Fig. 2 shows that the slope of the realized allocation is identical to the slope of the initial voucher allocation, suggesting that the sharing happens primarily within quantile of the PCF rather than across.

#### 4.3.2 Error rates

Table 2 shows the average village error rate (averaging first over individuals within villages, and then across villages) under the two allocation schemes (chiefs and PMT). For these calculations, we include only those villages in which the probability of getting a subsidy is between 0 and 100% (so that targeting errors are possible).^27^ The poverty-targeting error rate is the probability that a household is (1) eligible based on its position in the PCF distribution within the village; but (2) does not make it onto the actual beneficiary list (chief error) or on the counterfactual PMT beneficiary list (PMT error). Note that since the number of beneficiaries within the village is kept fixed in this exercise, this error rate also provides the probability that a household is (1) categorized as ineligible based on its position in the PCF distribution and (2) gets the subsidy. In other words, mechanically there are as many people who don’t get the subsidy when they should (exclusion errors) as there are people who get the subsidy when they should not (inclusion errors). We also show what the expected error rate would be if subsidies were allocated randomly. These are calculated from a permutation test with 1000 draws. Finally, we also compute the squared error for each allocation.

**Table 2:** Targeting errors: Comparison of chief and PMT allocations with consumption-based allocation.

	(1)	(2)	(3)	(4)	(5)	(6)
	Realized allocation of goods			Initial allocation of vouchers		
	2011 input subsidy	2012 input subsidy	2012 food subsidy	2011 input subsidy	2012 input subsidy	2012 food subsidy
Percentage of population receiving subsidy	0.768	0.802	0.586	0.527	0.545	0.343
Percentage of villages in which 0% received subsidy	0.000	0.000	0.049	0.000	0.000	0.082
Percentage of villages in which 100% received	0.098	0.164	0.049	0.033	0.016	0.016

*If between 0 and 100%*						
Simple error rate under following allocation mechanism:^a^						
Chief (true allocation)	0.158	0.144	0.151	0.223	0.217	0.178
PMT (counterfactual)	0.103	0.109	0.137	0.151	0.152	0.150
PMT (counterfactual) based on IHS3 formula	0.121	0.126	0.145	0.175	0.163	0.170
Random (counterfactual)	0.145	0.129	0.162	0.218	0.218	0.188
*P-val Chiefs=PMT*	<.001	0.004	0.221	<.001	<.001	0.009
*P-val Chiefs=PMT (IHS3)*	0.003	0.095	0.585	<.001	<.001	0.431
*P-val Chiefs=Random*	0.277	0.165	0.252	0.592	0.906	0.272
*P-val PMT=Random*	<.001	<.001	<.001	<.001	<.001	<.001

Mean squared error in log consumption under following allocation mechanism:^b^						
Chief (true allocation)	0.585	0.277	0.375	0.775	0.426	0.430
PMT (counterfactual)	0.168	0.129	0.160	0.337	0.208	0.245
PMT (counterfactual) based on IHS3 formula	0.325	0.161	0.250	0.507	0.255	0.388
Random (Counterfactual)	0.542	0.597	0.871	0.796	0.919	1.126
*P-val chiefs=PMT*	<.001	<.001	0.005	<.001	<.001	0.002
*P-val chiefs=PMT (IHS3)*	0.003	0.009	0.057	0.003	<.001	0.517
*P-val chiefs=Random*	0.591	<.001	<.001	0.800	<.001	<.001
*P-val PMT=Random*	<.001	<.001	<.001	<.001	<.001	<.001

Notes: IHS3 = Malawi Third Integrated Household Survey, a representative survey conducted by Malawi's National Statistical Office from March 2010 to March 2011.

a Error rate is defined as the percentage of people who received the subsidy and shouldn’t have. Since the total number of beneficiaries is fixed, this error rate is equal to the percentage of people who didn’t receive the subsidy and should have.

b Mean squared error is calculated as deviations from the log PCF threshold.

We can see that both allocations make a significant number of errors compared to the PCF-based allocation, but that the PMT always has a lower error rate. The error rates for chiefs is 15.8% and 14.4% for the 2011 and 2012 input subsidies, while the PMT's error rate is only 10.3% and 10.9%. For the food subsidy, the chief's error rate is 15.1%, compared to 13.7% for the PMT. Since not all errors are equally important (i.e. denying a subsidy to somebody just barely under the threshold is not nearly as costly as denying a very poor person), a more informative measure of errors may might be the mean-squared error (shown in the bottom of the table). Here too we see consistent evidence that the PMT has a lower MSE than do chiefs, across all subsidies types. Finally, we see that the PMT based on our data does consistently better than that based on the IHS3; however, both outperform the chiefs' allocation.

While chiefs do worse than the PMT, they do better than random (see Table 4). For the input subsidy, the simple error rate for chiefs is not statistically distinguishable from random, but the mean squared error is much lower, suggesting that chiefs trade PCF eligible for ineligible only around the PCF cutoff. Chiefs also do better than random on the food subsidy, by both metrics (Table 2).

**Table 3A:** Multivariate correlates of subsidy receipt (realized allocation).

	(1)	(2)	(3)	(4)	(5)	(6)	(7)	(8)
	Realized allocation				Counterfactual PMT allocation			
	Got input subsidy	Value (USD)	Got food subsidy	Value (USD)	Eligible for input subsidy	Value (USD)^a^	Eligible for food subsidy	Value (USD)^a^
Log PCF (total non-staple food expenditures per capita in past month)	−0.01	−0.31	−0.03**	−2.12*	−0.05***	−5.38***	−0.06***	−5.21***
	(0.01)	(0.62)	(0.01)	(1.21)	(0.01)	(0.53)	(0.01)	(0.94)

*Time-invariant baseline variables*								
Related to chief	0.06**	3.22*	0.12***	10.87***	−0.01	3.52***	0.02	2.31
	(0.02)	(1.71)	(0.03)	(2.89)	(0.02)	(1.27)	(0.02)	(1.61)
Log(acres farmed)	0.04***	5.40***	0.02	1.90	−0.05***	−5.99***	−0.07***	−8.54***
	(0.02)	(1.14)	(0.02)	(1.48)	(0.02)	(1.07)	(0.02)	(1.09)
Years of education (divided by 10)	0.02	2.26	−0.06	−3.38	−0.28***	−26.49***	−0.26***	−26.72***
	(0.03)	(2.65)	(0.05)	(4.10)	(0.03)	(2.42)	(0.04)	(2.99)
Widowed or divorced female	0.02	0.73	0.00	1.94	0.01	6.61***	0.07**	9.76***
	(0.03)	(1.60)	(0.03)	(2.88)	(0.02)	(1.66)	(0.03)	(2.04)
Household size (divided by 10)	0.07	8.27*	−0.04	−0.69	0.40***	48.33***	0.50***	71.19***
	(0.06)	(4.21)	(0.05)	(5.55)	(0.06)	(4.85)	(0.07)	(8.26)
Respondent age: 2nd quartile (26–35)	0.10***	8.80***	0.06*	4.32	0.05	3.45**	0.08**	1.88
	(0.03)	(2.42)	(0.03)	(3.04)	(0.04)	(1.71)	(0.03)	(2.36)
Respondent age: 3rd quartile (36–51)	0.15***	13.33***	0.12**	11.34***	0.10**	6.99***	0.10**	5.71*
	(0.04)	(2.90)	(0.05)	(3.96)	(0.04)	(2.40)	(0.04)	(2.93)
Respondent age: highest quartile (over 52)	0.19***	15.35***	0.24***	22.01***	0.14***	17.18***	0.21***	25.24***
	(0.04)	(2.79)	(0.05)	(4.33)	(0.04)	(2.79)	(0.04)	(3.22)
Log(value of animals owned)	0.00	1.12*	−0.01	0.74	−0.04***	−3.19***	−0.04***	−3.73***
	(0.01)	(0.67)	(0.01)	(0.88)	(0.01)	(0.58)	(0.01)	(0.86)

*Shocks*								
Experienced drought or flood (past 3 months)	0.03	−1.11	0.08**	5.45*	0.04	−0.87	0.03	3.20
	(0.02)	(1.50)	(0.03)	(2.74)	(0.02)	(1.61)	(0.03)	(2.55)
Experienced cattle death or crop disease (past 3 months)	0.05***	−2.16	0.00	0.21	0.03	−3.29**	0.02	0.93
	(0.01)	(1.45)	(0.02)	(2.21)	(0.02)	(1.42)	(0.02)	(1.74)
Number of observations	3118	3043	1559	1559	3118	3043	1559	1559
Number of households	1559	1558	1559	1559	1559	1558	1559	1559
Number of villages	61	61	61	61	61	61	61	61
Mean of dependent variable	0.79	50.47	0.59	42.03	0.79	50.47	0.59	42.03
Years	2011 & 2012		2012	2012	2011 & 2012		2012	2012

Note: Regressions for input subsidies pool years 2011 and 2012 and control for the year. Omitted age category is less than 26. Standard errors clustered at the village level. All regressions include village fixed effects.

a Counterfactual quantities have the same distribution as actual quantities.

* Significant at 10%.

** Significant at 5%.

*** Significant at 1%.

**Table 3B:** Multivariate correlates of subsidy receipt (initial allocation of vouchers).

	(1)	(2)	(3)	(4)
	Initial voucher allocation			
	Got input subsidy	Value (USD)	Got food subsidy	Value (USD)
Log PCF (total non-staple food expenditures per capita in past month)	0.00	0.18	−0.02*	−4.27*
	(0.01)	(0.67)	(0.01)	(2.23)

*Time-invariant baseline variables*				
Related to chief	0.06**	3.23	0.08***	15.10**
	(0.03)	(2.59)	(0.03)	(6.66)
Log(acres farmed)	0.06***	4.94***	−0.01	−0.92
	(0.02)	(1.58)	(0.02)	(3.56)
Years of education (divided by 10)	0.02	1.58	−0.06	−8.06
	(0.04)	(3.13)	(0.05)	(7.99)
Widowed or divorced female	0.04	4.03*	0.04	4.64
	(0.03)	(2.30)	(0.03)	(5.99)
Household size (divided by 10)	0.19***	15.79**	0.03	15.21
	(0.07)	(6.02)	(0.08)	(14.08)
Respondent age: 2nd quartile (26–35)	0.11***	10.35***	0.09***	7.99
	(0.03)	(2.85)	(0.03)	(5.25)
Respondent age: 3rd quartile (36–51)	0.22***	19.29***	0.18***	26.82***
	(0.05)	(3.89)	(0.04)	(7.72)
Respondent age: highest quartile (over 52)	0.36***	30.56***	0.34***	54.48***
	(0.05)	(4.19)	(0.04)	(8.99)
Log(value of animals owned)	0.02	0.98	0.00	1.75
	(0.01)	(0.93)	(0.01)	(2.11)

*Shocks*				
Experienced drought or flood (past 3 months)	0.08**	8.32***	0.07**	9.08
	(0.03)	(2.99)	(0.03)	(6.46)
Experienced cattle death or crop disease (past 3 months)	0.00	1.97	0.00	2.43
	(0.02)	(1.85)	(0.03)	(4.77)
Number of observations	3118	3043	1559	1559
Number of households	1559	1558	1559	1559
Number of villages	61	61	61	61
Mean of dependent variable	0.54	44.17	0.34	50.94
Years	2011 & 2012		2012	2012

Note: Regressions for input subsidies pool years 2011 and 2012 and control for the year. Omitted age category is less than 26. Standard errors clustered at the village level. All regressions include village fixed effects.

* Significant at 10%.

** Significant at 5%.

*** Significant at 1%.

**Table 4:** Productive efficiency.

	(1)	(2)	(3)	(4)	(5)	(6)	(7)
	Actual (chief's) allocations			Counterfactual PMT allocation			Ever lobbied chief to try to get input subsidy
	Value (USD) of input subsidy	Value (USD) of food subsidy	Value gap (input-food)	Value (USD) of input subsidy under PMT^a^	Value (USD) of food subsidy under PMT^a^	Value gap (input-food) under PMT	
*Panel A. Realized allocation of goods*							
Log(gain in farm production from fertilizer use)	4.08**	0.23	8.16**	−3.21	−4.13	1.73	0.00
	(1.70)	(2.93)	(3.44)	(2.68)	(3.28)	(3.14)	(0.03)
Log(total non-staple food expenditures per capita in past month)	−0.50	−0.58	0.23	−10.92***	−11.17***	2.05	0.01
	(0.75)	(1.57)	(2.14)	(1.10)	(1.66)	(1.51)	(0.01)

*Time-invariant baseline variables*							
Related to chief	2.32	10.74***	−7.27	9.03***	8.43**	−1.41	0.03
	(2.96)	(4.02)	(5.37)	(2.67)	(3.89)	(3.73)	(0.03)
Log(acres farmed)	6.26**	−1.28	6.28	−2.50	−5.20**	2.06	0.00
	(2.59)	(2.87)	(3.77)	(2.40)	(2.57)	(1.88)	(0.03)
Mean of dependent variable	51.83	37.78	11.94	53.19	39.42	10.62	0.089

*Panel B. Initial allocation of vouchers*							
Log(gain in farm production from fertilizer use)	6.07*	2.03	7.01	−4.29	−5.49	2.28	
	(3.11)	(5.83)	(6.13)	(3.55)	(6.63)	(6.04)	
Log(total non-staple food expenditures per capita in past month)	−3.22***	−4.10	0.19	−13.51***	−20.45***	6.65*	
	(1.12)	(3.29)	(3.47)	(1.15)	(4.23)	(3.67)	

*Time-invariant baseline variables*							
Related to chief	−1.44	17.49**	−18.32**	12.28***	12.96*	−2.45	
	(4.01)	(7.03)	(8.38)	(3.96)	(7.41)	(5.99)	
Log(acres farmed)	10.37***	−3.02	12.52*	−3.83	−6.86	2.81	
	(3.05)	(5.08)	(6.44)	(3.17)	(6.14)	(5.42)	
Mean of dependent variable	46.15	44.62	3.75	47.15	45.20	4.52	
Number of observations	1048	530	529	1048	530	529	
Number of households	530	530	529	530	530	529	
Number of villages	61	61	61	61	61	61	
Years	2011–2012	2012	2012	2011–2012	2012	2012	

Note: Sample restricted to households surveyed in 2014 and asked about perceived returns to fertilizer use. Regressions for input subsidies pool years 2011 and 2012 and control for the year. 2011 input allocation information comes from 2011 survey. 2012 input and food allocations information comes from 2012 survey. Omitted age category is less than 26. Standard errors clustered at the village level. All regressions control for village fixed effects.

a Counterfactual quantities have the same distribution as actual quantities.

* Significant at 10%.

** Significant at 5%.

*** Significant at 1%.

An interesting pattern in these results is that, compared to the PMT, chiefs look worse at targeting the truly needy for the input subsidy than for the food subsidy. A central hypothesis of this paper is that this may be due to productivity targeting of the input, which we will argue is less relevant for food. We dive into this issue in detail in Section 5.

#### 4.3.3 Who is favored and who is left out by chiefs?

Table 3A shows the results of a multivariate regression of the realized allocation (i.e. receiving a voucher or a share of a voucher) on background characteristics and village fixed effects. Columns 1–4 show regressions for the real-life allocation (decided by chiefs) while Columns 5–8 show a counterfactual allocation if the subsidies were allocated by the PMT formula. Table 3B performs the same analysis, but for receiving the voucher itself (i.e. not including people who received the subsidy via sharing). Comparing the coefficient estimates across the three sets of analyses tells us who is favored and who is left out under each scheme. We consider both the extensive margin (receiving any subsidy) and the intensive margin (the value of the subsidy received, since this varies across households due to sharing).^28^ The first row of Table 3A confirms the poverty-targeting results discussed above: the gradient in PCF is more negative under the PMT than under the chiefs, and the gap in the gradient is more pronounced for the input subsidy than for the food subsidy.

We find evidence of nepotism: conditional on covariates, chief's kin are 11 percentage points more likely to receive the food subsidy under the chief, whereas they would not be favored under the PMT. For the input subsidy, nepotism appears much less pronounced: while chief's kin receive a greater input subsidy package (an extra 3.30 kg compared to a mean of 50.5 kg, significant at 10%), the PMT would also award kin higher subsidy packages (+2.3 kg, also significant at the 10% level). This is due to the fact that chiefs' kin are marginally asset poorer than non-relatives. Turning to other covariates, we find that chiefs target older households, as per the official FISP recommendation. Chiefs also target households that received negative shocks: households who experienced a drought or flood are 4 percentage points more likely to receive subsidized food, while households who experienced crop loss or cattle death are 8 points more likely to get it. By contract, the PMT is not designed to respond to shocks, and indeed we find no correlation between shocks and subsidy receipt in the PMT (such a correlation might exist if shocks are strongly correlated with asset poverty).

### 4.4 Discussion of poverty-targeting results

The results in Tables 3A and 3B epitomize the trade-off between local information and capture: we find that chiefs are able to use local knowledge to benefit households hit by recent negative shocks, while the PMT misses them; but they also favor their kin. These results raise several questions.

First, is the fact that kin are more likely to get subsidies evidence of nepotism? An alternative hypotheses is that chiefs have better information on relatives, and therefore are more likely to target kin because they can be certain that they are truly poor. If this is the case, we would expect that subsidies to kin would be more responsive to consumption than to non-relatives. We investigate this in Table A4, in which we include an interaction between log food and kinship. We find no evidence in favor of the information hypothesis: targeting actually appears somewhat worse for relatives for the input subsidy, though there is no effect for the food subsidy. We also do not find that targeting based on shocks is better among relatives (Table A4), consistent with the fact that kin are favored irrespective of whether they faced a shock. While we lack data to definitively rule out an information story, our evidence appears more consistent with nepotism.

Second, how important is the mistargeting to relatives? Alatas et al. (2013) show that the “cost” of nepotism in terms of average consumption level among beneficiaries can be approximated with the following formula:

$$△C=\alpha \frac{△\beta }{\beta }\frac{\left(c_{e}-c_{b}\right)}{c_{b}}$$

where *α* is the share of kin, $\frac{△\beta }{\beta }$ is how much more likely kin are to receive benefits, and $\frac{\left(c_{e}-c_{b}\right)}{c_{b}}$ is how much richer kin are. Taking the following values from our data: $\alpha =0.27,\frac{△\beta }{\beta }=0.19$ (for the food subsidy)^29^ and $\frac{\left(c_{e}-c_{b}\right)}{c_{b}}=0.053$, we obtain △*C* ≈ 0.0027. In other words, because of nepotism, beneficiaries of the food subsidy have about 0.26% higher per capita expenditure than they would if nepotism were eliminated. This is a very small cost, in fact surprisingly similar to that obtained by Alatas et al. (2013) for Indonesia (△*C* ≈ 0.003 for a cash transfer program). There are two main reason why nepotism is not very costly in our setting. The first is that kin and non-kin are similarly poor. The second is that nepotism is relatively limited — many relatives are left out of the food subsidy, while many non-relatives are included. We calculate a counterfactual in which we re-allocate subsidies such that chiefs take away subsidies from non-relatives, giving them to relatives (taking the subsidies away from the richest non-relatives first, and prioritizing the poorest relatives). In this counterfactual, only 16% of non-relatives get subsidies, and the regression-adjusted increase for relatives becomes 48 percentage points, so that $\frac{△\beta }{\beta }=3$ and thus the welfare cost increases by a factor of 15.^30^

## 5 Productive efficiency targeting

In this section, we investigate whether some of the apparent mistargeting of input subsidies by chiefs is due to targeting on farming productivity: if returns to input subsidies are heterogeneous and chiefs have information on this, then they might allocate subsidies in a way that takes both poverty-targeting and productive efficiency into account. We use a simple model that allows for heterogeneity in returns as well as heterogeneity in the welfare weights that chiefs assign to households, to derive a test of whether the mistargeting we observe for input subsidies is in part driven by productive efficiency considerations.

### 5.1 Model and prediction

We consider the problem of allocating subsidies across households within a village. The intra-village allocation is done by the village chief.

Suppose that allocation of subsidy *s*_*i*_ (*s* ∈ {fertilizer, food}) to household *i* enables that household to generate additional income:

$$y_{i}=A_{is}s_{i}^{\mu }$$

where *A*_*i*_ denotes individual-specific returns to the subsidized resource and *μ* ∈ (0,1) denotes potentially diminishing returns to the subsidized resource. In the nested special case where the subsidized resource is food, rather than farming inputs, we set *μ* = 1 and *A*_*is*_ = 1 for all households (and thus start by abstracting away from a case in which there is a productive response to nutrition — we relax this assumption later).

We assume that households share a common homothetic, CRRA utility function defined over total income:

$$u_{i}=\frac{{\left(y_{i}+e_{i}\right)}^{1-\rho }}{1-\rho }$$

with *ρ* > 0,≠1 and where *e*_*i*_ is the income that household *i* gets in addition to the subsidy-enabled income.

The aggregate supply of subsidies to the village is denoted by $\bar{s}$. Under a proxy-mean test, the subsidies would go to the $\bar{s}$ households in the village with the lowest PMT score. In contrast, when allocating subsidies across households within the village, and assuming for now that there is no *ex post* income/output redistribution orchestrated by the chief, the chief chooses the subsidy levels *s*_*i*_ so as to maximize the weighted sum of villagers' utility:

(1) $$\sum \omega_{i}\frac{{\left(A_{is}s_{i}^{\mu }+\hat{e_{i}}\right)}^{1-\rho }}{1-\rho }$$

subject to

$$\underset{i}{\sum }s_{i}=\bar{s}$$

In Eq. (1), $\hat{e_{i}}$ is the income that the village chief expects household *i* to have at the time the subsidy benefits are realized, and *ω*_*i*_ is the relative welfare weight of household *i*. Since chiefs do not face reelection incentives and have limited accountability (see Section 2.1), the relative welfare weight of a household may not reflect its role in the political process as in earlier models (Bardhan and Mookherjee, 2000, Bardhan and Mookherjee, 2005, Bardhan and Mookherjee, 2006) but may instead depend on the preferences of the chief (e.g. if the chief favors his kin, the relative welfare weight of kin will be higher).

While ${ê}_{i}$ could be endogenous, we assume that the chief can take the households' best response distribution of $\hat{e_{i}}$ as given when maximizing the objective function shown in Eq. (1).

Taking the first order conditions for input subsidies (*s* = *fert*) for two households *i* and *j* yields:

(2) $$\omega_{i}{\left(A_{i}fert_{i}^{\mu }+{ê}_{i}\right)}^{-\rho }A_{i}fert_{i}^{\mu -1}=\omega_{j}{\left(A_{j}fert_{j}^{\mu }+{ê}_{j}\right)}^{-\rho }A_{j}fert_{j}^{\mu -1}$$

For food subsidies, where *A* = 1 and *μ* = 1 for all households, we have an analogous but simplified expression:

(3) $$\omega_{i}{\left(food_{i}+{ê}_{i}\right)}^{-\rho }=\omega_{j}{\left(food_{j}+{ê}_{j}\right)}^{-\rho }$$

Taking the ratio of Eq. (2) over Eq. (3), the welfare weights cancel and we obtain

(4) $$\frac{{\left(A_{i}fert_{i}^{\mu }+{ê}_{i}\right)}^{-\rho }A_{i}fert_{i}^{\mu -1}}{{\left(food_{i}+{ê}_{i}\right)}^{-\rho }}=\frac{{\left(A_{j}fert_{j}^{\mu }+{ê}_{j}\right)}^{-\rho }A_{j}fert_{j}^{\mu -1}}{{\left(food_{j}+{ê}_{j}\right)}^{-\rho }}$$

**Fig. A1:**
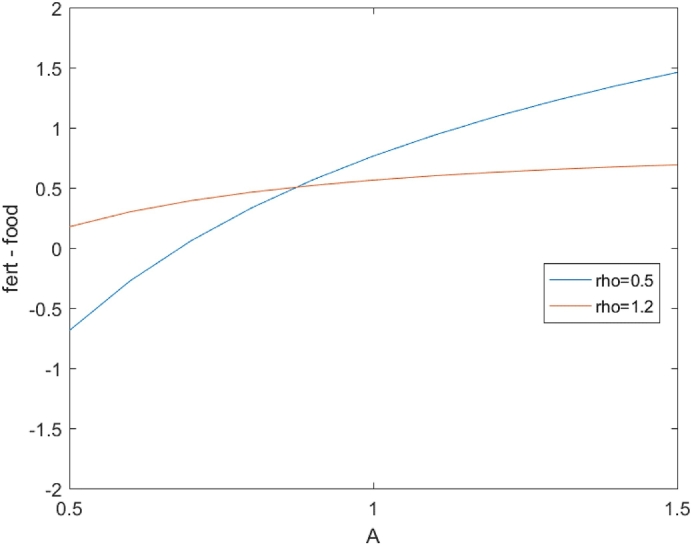
Model simulation: Optimal allocation with productive efficiency consideration. Notes: see text in Section 5.

From this expression we can derive the relationship between a household's productivity parameter *A*_*i*_ and the difference in value between the fertilizer and the food subsidy that household receives $\left(fert_{i}-food_{i}\right)$. In Fig. A1, we plot that relationship setting *μ* = 0.9 and either *ρ* = 0.5 or *ρ* = 1.2. The relationship is positive: as the returns to fertilizer increase, a household receives relatively more fertilizer subsidies than food subsidies. The intuition here is the following: if productivity considerations matter, then if a household has a higher return to the fertilizer subsidy than average, then that household should be relatively more favored when it comes to the input subsidy than for the food subsidy. Unsurprisingly, when the utility function is very concave (*ρ* = 1.2), productive efficiency considerations are considerably muted, since increases in the resources of the already better off have lower value.

This leads us to the prediction we can test in the data:

Prediction 1 If chiefs take into consideration productive efficiency when allocating farming subsidies, $d\left(fert_{i}-food_{i}\right)/dA_{i}>0$. Namely, the higher the return to fertilizer for a household, the higher the gap between fertilizer and food subsidies received by that household.

#### 5.1.1 Allowing chiefs to orchestrate transfers

As shown in Table 1 Panel C, there is a significant amount of transfers between households within the village. In the presence of a redistribution instrument, the chief's objective function would be modified as follows: the chief now chooses the sets of subsidies *s*_*i*_ and transfers *t*_*i*_ so as to maximize

$$\underset{i}{\sum }\omega_{i}\frac{{\left(A_{i}s_{i}^{\mu }+t_{i}+{ê}_{i}\right)}^{1-\rho }}{1-\rho }$$

subject to

$$\begin{array}{lll} \underset{i}{\sum }s_{i} & =\bar{s} & \\ \underset{i}{\sum }t_{i} & =0 & \end{array}$$

where *t*_*i*_ is the net ex post income transfer received by household *i*, which can be either negative or positive.

It is evident that redistribution will allow chiefs to target productivity more than the autarkic case. Thus the more redistribution is possible, the greater the optimal wedge between the fertilizer and the food subsidy a given household receives. In the extreme case in which income is fully pooled, the objective function of the chief can be rewritten as $\max\sum_{i}\beta_{i}\left(A_{i}s_{i}^{\mu }\right)$. In this case, the allocation of fertilizer subsidies will be entirely driven by productive efficiency since redistribution will happen ex post.

Prediction 2 Productive efficiency considerations when allocating farming subsidies increase as the level of ex post income-pooling in the village increases.

Prediction 2 brings attention to the fact that the two subsidies we study could be complementary: the input subsidy as a growth instrument and the food subsidy as a redistribution instrument. This logic could also imply that the food subsidy and input subsidy allocations could be related — farmers who received the input subsidy should have larger harvests and be less in need of the food subsidy. Note that this does not invalidate our test: the food subsidy should be allocated based on Pareto weight and current consumption, irrespective of whether the current consumption level was secured through enhanced yields in the previous period thanks to inputs subsidies or not. Relative Pareto weights can still be backed out from jointly observing the food allocation and current consumption, as we do.^31^

Below we show that our predictions hold under a number of extensions to the basic model.

#### 5.1.2 Productive response to better nutrition

It is possible that food subsidies increase productivity for very poor households due to improved nutrition (Strauss, 1986). Such a nutrition-productivity link would not change the predictions. To see this, note that allowing for the efficiency of an hour worked to increase with food subsidies implies a negative correlation between the relative productivity of inputs and the relative productivity of food, given the complementarity between farm inputs and efficient labor units. This increases $d\left(fert-food\right)/dA$.

#### 5.1.3 Price effects

In many African countries, rural economies are poorly connected to markets and thus local prices may be responsive to local output. If so, allocating subsidies to the most productive may reduce prices by increasing output. Since 90% of farmers in our sample are *net buyers* of grain (consuming more grain than they produce), such a price effect would translate into a positive income effect for most villagers and thereby increase welfare. This increases $d\left(fert-food\right)/dA$ for any *ρ* because allocating inputs to households with higher returns increases the welfare of the rest of the village through lower prices.

### 5.2 Results

To test predictions, we need a measure of *A*, the household-specific (farm-specific) productivity of fertilizer. In this or any context, estimating the productivity of an input is very difficult, since input choices are endogenous and farmers with higher returns are presumably more likely to use fertilizer in a given season. Returns are also volatile across years and even within farms, so estimating this well would typically require a long panel.

Instead of estimating productivity, we therefore opted to simply ask farmers for their expectations of yields with and without fertilizer use. We collected this data in the fifth survey round conducted in the summer 2014. There are several important caveats. First, due to budget constraints the survey could only be done with a random subset of households in each village. The sample includes only about one third of the sample. Second, the questions are about total output with and without fertilizer, rather than marginal returns.^32^

We show the means of the reported expected yield in Panel C of Table 1, and we plot the distribution of the reported gain in total output in Panel B of Fig. W in the Web Appendix. There is substantial heterogeneity in these reported gains from input use. What drives it? Table A5 examines correlates of self-reported gains. We regress the reported log yield increase on log acres and other observables. We find that reported gains are correlated with many variables, including household demographics (gains are increasing in the age of the head of household), education, log assets, and household size (though this is not statistically significant). We expect that these are the types of proxies that the chief may use to target subsidies, in addition to other characteristics that are unobservable to us, such as land quality. Also of note is that the correlation between estimated production gains from using fertilizer and our measure of neediness, PCF, is fairly weak (Panel C of Fig. W). We also find no systematic differences by kinship status (Table 1 Panel C, column 3, and Table A5).

**Fig. 3:**
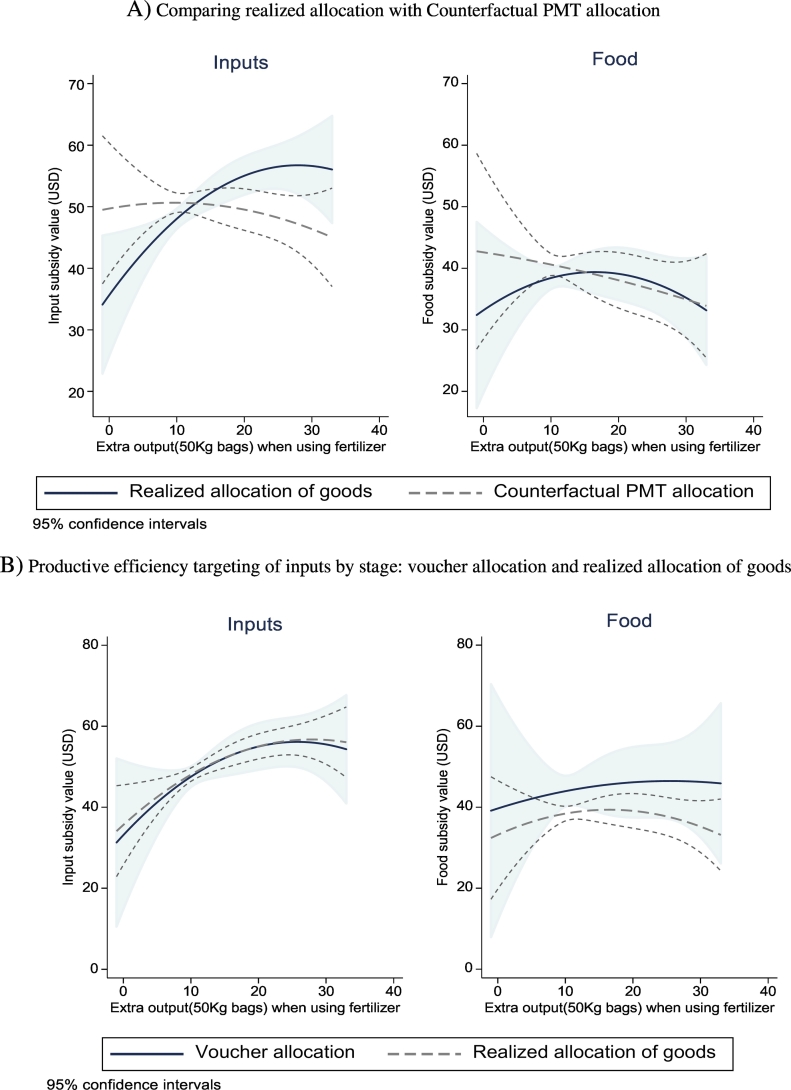
Productive efficiency of chief's allocation: polynomial estimates. Notes: 2012 data. Estimates from OLS regressions with second-order polynomial in the variable shown on the x-axis as well as controls for PCF, log land size, and chief kinship.

To test for productive efficiency targeting, in Table 4 we regress the value of the fertilizer and food subsidies received, as well as their gap $\left(fert_{i}-food_{i}\right)$, on the log of reported gains in output when using fertilizer. We find clear evidence in favor of targeting based on productive efficiency. The value of the input subsidy received increases significantly with the reported gains from fertilizer use. The food subsidy, by contrast, is not correlated with gains. When we look at our primary outcome — the gap between the two — we find that the gap increases significantly with the gain from fertilizer, as predicted by the model. In Fig. 3 we plot the estimated relationship between the subsidy values and the gain when using a quadratic instead of log. The positive slope for input subsidy values under the chief's allocation is very clear, compared to the flat relationship for food subsidies.

These results are in sharp contrast with those for the counterfactual PMT distribution, in which the value of the subsidy is actually (insignificantly) declining in the gains to fertilizer (because of a negative correlation between returns and assets). In that case, the PMT undermines the effect of the subsidy on total farm output at the village level. In contrast with the chief's allocation, the gap between fertilizer and food subsidy values does not significantly increase with reported gains from fertilizer under the PMT allocation (Table 4, column 6).^33^

Overall, the results in Table 4 and Fig. 3 are consistent with chiefs taking productive efficiency into consideration when allocating input subsidies — something that the PMT cannot do since information on who has more to gain from fertilizer use is not something that can be elicited in an incentive-compatible way if people expect their subsidy package to depend on it. The magnitude of the effects is not trivial: a household with an extra log point gain from fertilizer gets about 6.5 more kgs of input subsidies under the chiefs than under the PMT.

**Fig. 4:**
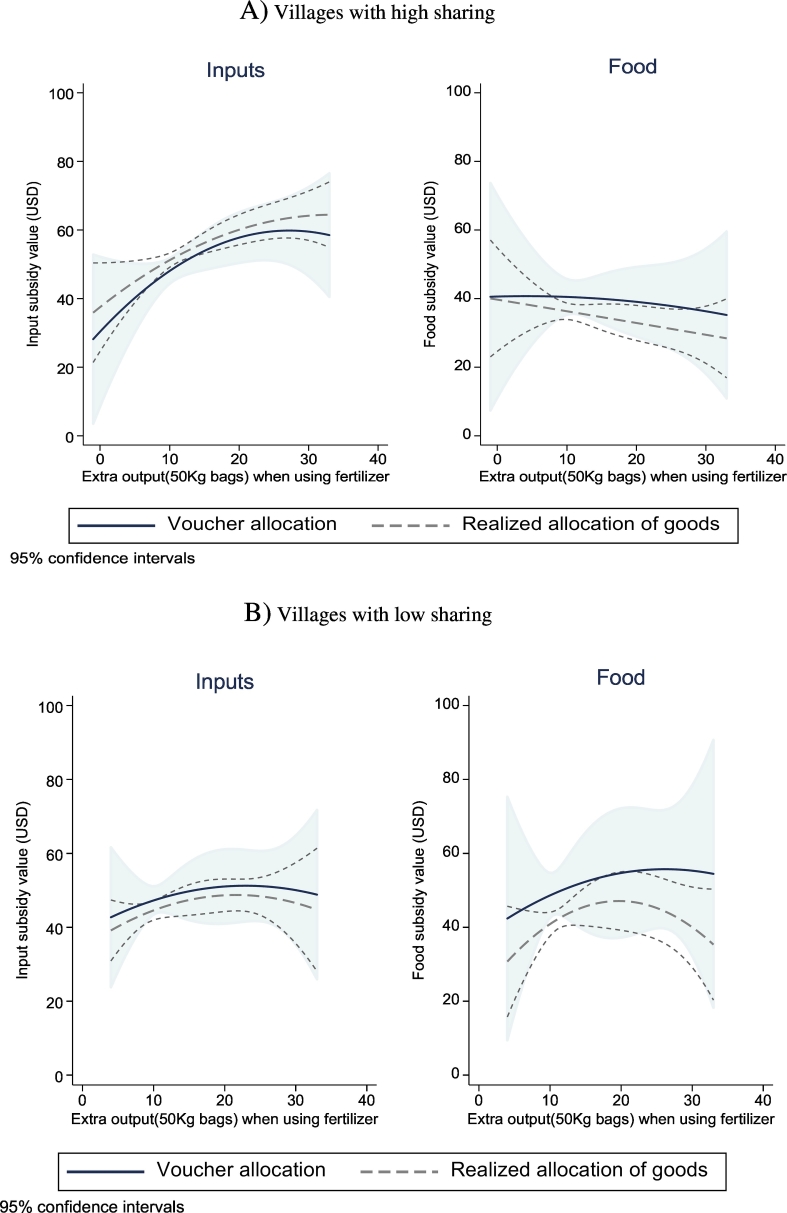
Productive efficiency targeting by village sharing level: polynomial estimates. Notes: 2012 data. High sharing villages are those where the number of transfers to other households, as a fraction of village size, is above the village-level median. Estimates from OLS regressions with second-order polynomial in the variable shown on the x-axis as well as controls for PCF, log land size, and chief kinship.

We test the second prediction in Table 5 and Fig. 4, which replicates the analyses in Table 4 and Fig. 3, but looking separately at villages where there appears to be more or less income-pooling than in the median village. Our measure of income-pooling at the village level is based on reported inter-household transfers. Namely, we asked all sampled households about transfers made to other villagers within the past 90 days. We take the village-level average and split the villages at the median. We call villages with an above-median number of transfers “high-sharing” and those below are labeled “low-sharing.” In Fig. 4, we find that the slope of the relationship between productivity and subsidy receipt is much steeper in villages with a higher degree of income-pooling, and Table 5 confirms that the slope $d\left(fert-food\right)/dA$ is statistically significantly higher in those villages as predicted. High levels of sharing allow chiefs to use the input subsidies as a growth instrument, bringing their village closer to the production possibility frontier. Interestingly, allocating input subsidies based on returns is not what they are asked to do. The official guidelines of the inputs subsidy program is to target the poor, and thus when asked chiefs report targeting the poor rather than taking productivity into account (see Table A3) — even though our careful analysis of their allocation decision suggests that they do.^34^

**Table 5:** Heterogeneity in efficiency and poverty-targeting.

	(1)	(2)	(3)	(4)	(5)	(6)			
	Villages with low sharing			Villages with high sharing			*P*-val low sharing = high sharing		
	Value (USD) of input subsidy	Value (USD) of food subsidy	Value gap (input-food)	Value (USD) of input subsidy	Value (USD) of food subsidy	Value gap (input-food)	Value (USD) of input subsidy	Value (USD) of food subsidy	Value gap (input-food)
*Panel A. Realized allocation of goods*									
Log(gain in farm production from fertilizer use)	3.82	4.70	0.28	4.07*	−3.73	14.05***	0.94	0.15	0.04
	(2.77)	(5.22)	(6.07)	(2.35)	(3.53)	(3.94)			
Log(total non-staple food expenditures per capita in past month)	−1.14	0.86	−1.75	0.10	−1.90	2.31	0.39	0.36	0.31
	(1.11)	(2.26)	(2.92)	(1.01)	(2.36)	(3.25)			
Related to chief	2.73	9.79*	−8.43	1.70	11.61**	−6.85	0.87	0.80	0.87
	(5.40)	(5.69)	(7.64)	(3.41)	(5.64)	(7.50)			
Mean of dependent variable	49.0	41.4	4.0	54.2	34.7	18.6			

*Panel B. Initial allocation of vouchers*									
Log(gain in farm production from fertilizer use)	4.47	7.01	−4.56	6.67	−2.96	16.05**	0.73	0.39	0.08
	(5.24)	(10.18)	(10.03)	(4.09)	(7.49)	(7.71)			
Log(total non-staple food expenditures per capita in past month)	−4.23**	−3.05	0.03	−2.17	−4.96	0.51	0.33	0.77	0.94
	(1.66)	(5.35)	(5.33)	(1.50)	(4.42)	(4.93)			
Related to chief	−1.29	17.40	−16.88	−1.70	18.63*	−20.21	0.96	0.93	0.83
	(7.21)	(11.40)	(11.50)	(4.75)	(9.17)	(11.99)			
Mean of dependent variable	45.1	50.5	−3.3	47.1	39.7	9.7			
Number of observations	480	242	242	568	288	287			
Number of households	242	242	242	288	288	287			
Number of villages	31	31	31	30	30	30			
Years	2011–2012	2012	2012	2011–2012	2012	2012			

Note: Sample restricted to households surveyed in 2014 and asked about perceived returns to fertilizer use. Regressions for input subsidies pool years 2011 and 2012 and control for the year. 2011 input allocation information comes from 2011 survey. 2012 input and food allocations information comes from 2012 survey. “Low Sharing” are villages with average transfers to nearby neighbors per household below the village-level median. Standard errors clustered at the village level. All regressions control for village fixed effects.

* Significant at 10%.

** Significant at 5%.

*** Significant at 1%.

### 5.3 Supportive evidence

Is information on the relative productivity of various potential beneficiaries of the input subsidy embedded in the chief, or does it rest in the people themselves? People who have high value for the input subsidy could wait in line more, lobby more or protest more if they don’t get the subsidy, such that the allocation of the chief ultimately favors them in a way that looks as if the chief himself were aware of the heterogeneity.

To provide descriptive evidence on this question, in the 2014 survey, we asked respondents if they had ever lobbied the chief to obtain subsidies. Only 9% of respondents reported lobbying for input subsidies, and 4% reported lobbying for food subsidies (Table W1). The likelihood of having lobbied is not positively correlated with returns to fertilizer for the overall sample (see Table 4, column 7), though it is correlated among the chiefs' kin (see Table A6, column 7). However, we argue that the this lobbying is of modest importance, since kin lobby much less on average, and overall the targeting efficiency is not higher among kin as shown in Table A6 columns 1–3.

In the survey of chiefs also conducted in 2014, we asked chiefs a number of questions about what they could observe about households, which we present in Web Appendix Table W7. We find that 86% of chiefs report that they can easily categorize farms in their village in terms of productivity of inputs. Chiefs also report that they know who works harder, who has money for inputs, and whose returns are highest. While descriptive, these responses are consistent with chiefs having significant local knowledge.

### 5.4 Threats to validity

In this section, we discuss several possible threats to internal validity: (1) the fact that returns to inputs are self-reported rather than observed, and (2) the fact that the two subsidy programs we consider exist alongside other social programs which may be allocated simultaneously.

#### 5.4.1 Using self-reported returns

A possible concern with our analysis is that returns are self-reported rather than directly observed, and so could potentially be correlated with various omitted variables. We present several pieces of evidence to help address this. First, we use our data to construct an agricultural panel. Specifically, we have complete data for the 2010–2011 and 2011–2012 planting seasons in this paper. From this we have at most 2 observations on households about their fertilizer use and output. We utilize this by running fixed effects regressions of output on input usage — relying on variation in input usage that occurs over years. The key variable in this regression is an interaction between self-reported gains and input usage — if the measure is valid, then this correlation should be positive. We show results in Table A7. We find strong evidence that these (admittedly non-random) returns to fertilizer are higher for those with higher self-reported returns. While we do not want to make too much of this since input usage is potentially endogenous, this fixed effects specification does rule out some time-invariant sources of bias — for example, land size and household demographics are held fixed in this analysis. At least descriptively, these results seem to support our interpretation.

Beyond these results, we argue that many (though not all) stories for why households might get more subsidies would apply to both food and fertilizer subsidies. For example, one might argue that people with higher returns are more confident and have higher social status and are therefore more likely to get subsidies. But these sorts of stories would not explain our results, since these households would get more fertilizer *and* food, whereas our main empirical tests are about the *difference* in the value of the two types of subsidies. We also find that this relationship is stronger in villages with higher levels of sharing. While this is consistent with the framework we have written down, it does not seem likely that we would observe this particular pattern (which was derived ex ante) if the results were driven purely by omitted variables.

#### 5.4.2 Other safety net programs

Beegle et al. (2017) document that chiefs are also involved in deciding which households are eligible for Malawi's public work program (PWP) — though the responsibility falls more on the Group Village Headmen and the villagers themselves. They report that Malawi's PWP “has been operational since the mid-1990s and aims to provide short-term labor-intensive activities to poor, able-bodied households for the purpose of enhancing their food security.” While we did not collect data on participation in the PWP directly from respondents in our surveys, a fuzzy name match between the original household sample and administrative data on PWP participants obtained from the two districts in our sample yields 167 matches for the 2012–2013 budget year, out of 2107 households in the DKRU baseline survey, suggesting that the PWP coverage in our study area is about 8%. Verification surveys with a subset of those matched and unmatched conducted in March 2015 suggests that an additional 3% may have been participating in PWP, bringing our estimates to roughly 11%.^35^ While name matching is always prone to significant error, this ballpark figure is not far from the 15% coverage targeted by the program. While studying how the PWP is targeted and the specific role of chiefs would have been interesting, omitting it due to data limitations should not affect our analysis of the other subsidy programs. Notably, Beegle et al. (2017) find no correlation between receipt of PWP and receipt of other benefits, suggesting no “fairness norm” influencing distribution across programs, in particular, no compensation of non-PWP households with input or food subsidies.

## 6 Conclusion

Traditional leaders, often known as “chiefs,” have maintained a significant amount of *de facto* if not *de jure* power in sub-Saharan Africa. Possibly owing to the weakness of local governance in most of the continent, chiefs are commonly involved in the decisions of how to allocate government resources. One prominent type of resource is subsidies. Developing country governments allocate an important portion of their national budget to subsidies targeted at the poor, and it is common for chiefs to be asked to identify who should be eligible for such subsidies. Do chiefs identify the right beneficiaries? Previous work on this question in Malawi concluded that there was widespread elite capture (Dorward et al., 2008, Kilic et al., 2013), based on evidence that “connected” households are more likely to receive subsidies, and that household assets measures do not strongly predict subsidy receipt. We show that such evidence may not directly speak to the issue of poverty-targeting in environments where assets are a poor predictor of need, and where the subsidized items are productive inputs.

We find evidence that chiefs allocate input subsidies to farmers with larger returns to input use. This result underscores how a naive measure of targeting based solely on the neediness of households (even when neediness is well measured) may understate the poverty-alleviation impacts of the allocation: when ex post redistribution is possible through informal transfers, targeting input subsidies based on productive efficiency (i.e. using input subsidies as a *growth instrument*) can have a larger impact on aggregate welfare than targeting based on poverty would. This issue has not received much attention in the literature up to this point, even though most of the inputs subsidized by governments are productive (farming inputs, health products) that have heterogeneous returns. Future work should explore whether our results generalize to other contexts and countries.

## Figures and Tables

**Table A1:** Summary statistics on villages and chiefs in the sample.

	(1)	(2)	(3)	(4)	(5)
	Villages (N = 61)				
	Mean	SD	High sharing	Low sharing	*P*-val high = low
*Panel A. Characteristics of villages*					
Village population	3169	4149	3412	2856	0.57
Number of households in village	309	333	339	269	0.39
Number of distinct family clans in village	63	186	44	87	0.36
Total acres of customary land in village	7640	7294	6955	8540	0.37
Village age	67	39	70	64	0.62
Village in Machinga district	0.48		0.63	0.32	0.02
Village wealth (mean asset holdings in 100 USD)	0.86	0.42	0.78	0.93	0.15
Village asset inequality (interquartile range in 100 USD)	0.85	0.39	0.74	0.95	0.03
Religious diversity index	0.31	0.17	0.31	0.30	0.77

	Village chiefs (N = 79)				
	Mean	SD	High sharing	Low sharing	*P*-val high = low

*Panel B. Characteristics of chiefs*					
Age	53.96	14.99	52.48	55.88	0.34
Male	0.82		0.82	0.83	0.91
Years of education	5.25	3.54	5.36	5.11	0.76
Religion					
Christian	0.39		0.23	0.60	<.001
Muslim	0.61		0.77	0.40	<.001
How were you selected to be chief?					
Hereditary	0.90		0.86	0.94	0.23
Appointed	0.09		0.14	0.03	0.08
Elected	0.01		0.00	0.03	0.32
For how many years have you been chief?	13.24	13.23	13.05	13.49	0.89
For how many years have you lived in this village?	44.90	17.25	43.43	46.74	0.40
For how many years have you farmed the land you currently farm?	24.19	14.70	22.91	25.80	0.40
At the time you became chief, was someone else considered for the position?	0.05		0.05	0.06	0.82
Do you receive a payment (*mswahala*) from the government?	0.89		0.89	0.89	0.99
Have you ever been suspended from your position as village head	0.01		0.02	0.00	0.32
*Describe your responsibilities as village chief*					
Solve conflicts among villagers	0.90		0.90	0.88	0.81
Report to group village headman and traditional authority	0.61		0.60	0.65	0.68
Monitor village projects	0.56		0.55	0.59	0.75
Disseminate information to villagers	0.33		0.35	0.32	0.81
Oversee subsidy programs	0.20		0.13	0.32	0.05
Preserve local traditions	0.14		0.13	0.15	0.79
Demarcate and supervise use of customary land	0.06		0.08	0.03	0.38
Supervise government laws	0.01		0.00	0.03	0.33

Notes: Data from surveys conducted from August to October 2014 in the study districts. High sharing villages are those where the number of transfers to other households, as a fraction of village size, is above the village-level median.

**Table A2:** Chiefs' role in the allocation of subsidies.

	(1)	(2)
	Input subsidy	Food subsidy
*Panel A. Surveys of chiefs in 2014 (N=79)*		
*Who decides which households in the village will be beneficiaries of the subsidy program?*		
Village head (chief) alone	0.62	
Village development committee alone	0.13	
Village meeting	0.13	
District agricultural officer alone	0.06	
Group village head alone	0.04	
Chief in consult with others	0.03	
*What selection criteria are used to allocate vouchers in your village? (multiple answers possible)*		
Neediness	0.97	1.00
Absence of male head	0.62	0.54
Recent negative shocks	0.54	0.34
Child headed households and households taking care of orphans	0.24	0.20
How hard-working the household is	0.16	–
Farm size	0.11	–
Elderly, disabled, or chronically ill	0.05	0.76
Land quality	0.01	–

*Panel B. Survey of villagers in 2013 (N=1381)*		
The chief organized a meeting to talk about the program	0.95	0.81
If yes, did you attend the meeting?	0.82	0.65
At the meeting, was there a discussion about:		
Who should be included in the program?	0.77	0.81
Sharing the subsidies (i.e. who should share with who, how much should be shared)?	0.75	0.71

*Panel C. Survey of villagers in 2014 (N=542)*		
Have you ever made a payment to the chief? (not specific to subsidy)	0.44	
*Who decides which households in the village will be beneficiaries of the subsidy program?*		
Village head (chief) alone	0.49	0.73
Chief in consult with others	0.23	0.04
Village meeting	0.15	0.02
Village development committee alone	0.10	0.09
District agricultural officer alone	0.01	–
NGO alone	–	0.08
Group village head alone	0.02	0.03
Other	0.01	0.02
*Have you ever asked the village head to give you an input subsidy voucher?*	0.09	0.03
*Have you ever complained to the village head about the allocation?*	0.16	0.05
*What selection criteria are used to allocate vouchers in your village?*		
Neediness	0.71	0.88
Elderly, disabled, or chronically ill	0.46	0.75
Child headed households and households taking care of orphans	0.16	0.29
Absence of male head	0.12	0.37
Recent negative shocks	0.10	0.34
How hard-working the household is	0.13	–
Farm size	0.01	–
Households with more children	–	0.32
Households with poor land	–	0.27
Households not receiving other subsidies	–	0.26

Notes: Panels A and C come from surveys administered in August–October 2014. Panel B comes from a survey administered Feb–May 2013.

**Table A3:** PMT formula.

	(1)	(2)
	Log PCF	Log PAEF
Household size (divided by 10)	−4.73***	−3.04***
	(0.74)	(0.55)
Household size (divided by 10) squared	1.91***	1.22***
	(0.48)	(0.42)
Number of children under 5 (divided by 10)	–	1.23***
		(0.34)
Total number of children (divided by 10)	0.54*	–
	(0.32)	
Log durable assets	0.40***	0.40***
	(0.03)	(0.03)
Log animal assets	–	–
Owns land	–	–
Owns land * log acres owned	0.10**	0.09**
	(0.04)	(0.04)
Widowed or divorced female head	−0.27***	–
	(0.07)	
Age of respondent (divided by 100)	–	–
Age of respondent (divided by 100) squared	−1.28***	−1.30***
	(0.28)	(0.27)
Highest education within household	0.05***	0.06***
	(0.01)	(0.01)
Household head is literate	–	–
Home has mud or dirt floors	−0.16*	–
	(0.10)	
Home has thatch roof	–	–
Home has mud or dirt walls	–	–
Toilet is private covered latrine	–	–
Toilet is uncovered latrine	–	–
No toilet	–	–
Water source is public tap	0.69***	0.64***
	(0.19)	(0.20)
Water source is well	0.58***	0.55**
	(0.19)	(0.21)
Water source is piped water	1.02***	1.05***
	(0.32)	(0.30)
Has electricity	–	–
Has a mobile phone	–	–
Main occupation = vendor	–	–
Main occupation = owner of other business	–	–
R-squared	0.32	0.28
Households	1559	1559
Villages	61	61

Notes: Baseline data. PC(PAE)F = per capita (per adult eq.) expenditures on non-staple food (monthly eq.), in USD. Sequential selection of variables done using Stata backward step-wise regression. Standard errors, clustered by village, in parentheses.

* Denotes significance at 10%.

** Denotes significance at 5%.

*** Denotes significance at 1%.

**Table A4:** Is poverty-targeting more efficient among chiefs' kin?

	(1)	(2)	(3)	(4)	(5)	(6)	(7)	(8)
	Realized allocation of goods (chiefs)				Counterfactual PMT allocation			
	Got input subsidy	Value (USD)	Got food subsidy	Value (USD)	Eligible for input subsidy	Value (USD)^a^	Eligible for food subsidy	Value (USD)^a^
Log PCF (total non-staple food expenditures per capita in past month)	−0.01	−0.81	−0.03**	−1.78	−0.06***	−5.69***	−0.05***	−5.17***
	(0.01)	(0.67)	(0.01)	(1.35)	(0.01)	(0.56)	(0.01)	(1.14)
Log PCF * related to chief	0.01	1.90*	0.00	−0.85	0.02	1.25	−0.02	0.00
	(0.02)	(1.11)	(0.03)	(2.67)	(0.01)	(0.88)	(0.01)	(1.59)

*Time-invariant baseline variables*								
Related to chief	0.06**	2.89	0.13***	10.12***	−0.02	2.59	0.03	1.01
	(0.02)	(1.98)	(0.03)	(3.28)	(0.02)	(1.59)	(0.03)	(2.33)
Log(acres farmed)	0.04***	5.40***	0.02	1.91	−0.05***	−5.99***	−0.07***	−8.53***
	(0.02)	(1.13)	(0.02)	(1.45)	(0.02)	(1.07)	(0.02)	(1.09)
Years of education (divided by 10)	0.02	2.40	−0.06	−3.87	−0.28***	−26.41***	−0.26***	−26.84***
	(0.03)	(2.65)	(0.05)	(4.10)	(0.03)	(2.41)	(0.04)	(3.00)
Widowed or divorced female	0.02	0.77	0.00	1.85	0.01	6.62***	0.07**	9.71***
	(0.03)	(1.60)	(0.03)	(2.79)	(0.02)	(1.65)	(0.03)	(2.04)
Household size (divided by 10)	0.07	8.34*	−0.04	−0.60	0.40***	48.41***	0.50***	71.24***
	(0.06)	(4.25)	(0.05)	(5.52)	(0.06)	(4.82)	(0.07)	(8.29)
Respondent age: 2nd quartile (26–35)	0.10***	8.68***	0.06*	4.51	0.05	3.37*	0.08**	1.90
	(0.03)	(2.42)	(0.03)	(3.05)	(0.04)	(1.70)	(0.03)	(2.34)
Respondent age: 3rd quartile (36–51)	0.15***	13.19***	0.12**	11.64***	0.10**	6.95***	0.10**	5.83**
	(0.04)	(2.94)	(0.05)	(3.96)	(0.04)	(2.40)	(0.04)	(2.89)
Respondent age: highest quartile (over 52)	0.19***	15.29***	0.23***	22.01***	0.14***	17.18***	0.21***	25.28***
	(0.04)	(2.81)	(0.05)	(4.30)	(0.04)	(2.80)	(0.04)	(3.17)
Log(value of animals owned)	0.00	1.11	−0.01	0.72	−0.04***	−3.21***	−0.04***	−3.74***
	(0.01)	(0.67)	(0.01)	(0.88)	(0.01)	(0.58)	(0.01)	(0.86)

*Shocks*								
Experienced drought or flood (past 3 months)	0.03	−2.3	0.11**	9.23**	0.04*	−1.69	0.02	3.85
	(0.03)	(2.23)	(0.05)	(3.89)	(0.02)	(2.09)	(0.03)	(2.91)
Experienced cattle death or crop disease (past 3 months)	0.06***	−0.94	−0.01	−2.69	0.01	−3.65**	0.03	−0.49
	(0.02)	(1.64)	(0.03)	(2.49)	(0.02)	(1.57)	(0.03)	(2.08)
Related to chief * experienced drought or flood (past 3 months)	0.00	4.13	−0.12	−12.74	−0.03	3.07	0.03	−1.88
	(0.05)	(4.09)	(0.08)	(8.42)	(0.04)	(3.76)	(0.06)	(5.00)
Related to chief * experienced cattle death or crop disease (past 3 months)	−0.05	−4.05	0.02	9.45*	0.04	1.48	−0.01	4.98
	(0.03)	(2.75)	(0.06)	(4.97)	(0.04)	(2.50)	(0.05)	(4.06)
Number of observations	3118	3043	1559	1559	3118	3043	1559	1559
Number of households	1559	1558	1559	1559	1559	1558	1559	1559
Number of villages	61	61	61	61	61	61	61	61
Mean of dependent variable	0.79	50.47	0.59	42.03	0.79	50.47	0.59	42.03

Note: Regressions for input subsidies pool years 2011 and 2012 and control for the year. Omitted age category is less than 26. Standard errors clustered at the village level. All regressions include village fixed effects.

a Counterfactual quantities have the same distribution as actual quantities.

* Significant at 10%.

** Significant at 5%.

*** Significant at 1%.

**Table A5:** Multivariate correlates of returns to fertilizer.

	Dependent variable: Log(gain in farm production from fertilizer use)
Log(acres farmed)	0.13***
	(0.04)
Related to chief	−0.01
	(0.04)
Years of education (divided by 10)	0.25***
	(0.08)
Widowed or divorced female	−0.01
	(0.05)
Household size (divided by 10)	0.23*
	(0.12)
Respondent age: 2nd quartile (26–35)	0.13**
	(0.06)
Respondent age: 3rd quartile (36–51)	0.21***
	(0.06)
Respondent age: highest quartile (over 52)	0.22***
	(0.08)
Log(value of animals owned)	0.06***
	(0.02)
Ever made a payment to the village chief	−0.01
	(0.05)
Number of observations	530
Number of villages	61
Mean of dependent variable	2.04
SD of dependent variables	0.74
R-squared (no village FE)	0.14

Note: Omitted age category is less than 26. Standard errors clustered at the village level. Regression includes village fixed effects.

* Significant at 10%.

** Significant at 5%.

*** Significant at 1%.

**Table A6:** Is productive efficiency targeting (post-sharing) stronger among chiefs' kin?

	(1)	(2)	(3)	(4)	(5)	(6)	(7)
	Actual (chief's) allocations			Counterfactual PMT allocation			Ever lobbied chief
	Value (USD) of input subsidy	Value (USD) of food subsidy	Value gap (input-food)	Value (USD) of input subsidy under PMT^a^	Value (USD) of food subsidy under PMT^a^	Value gap (input-food) under PMT	to try to get input subsidy
Log(gain in farm production from fertilizer use)	3.94*	−2.52	9.16**	−3.07	−3.25	0.40	−0.03
	(2.34)	(3.55)	(4.22)	(3.05)	(3.99)	(3.43)	(0.03)
Log(gain in farm production from fertilizer use) * relative	0.37	8.87	−3.37	−0.47	−2.71	4.13	0.10**
	(4.25)	(6.55)	(8.08)	(4.31)	(5.93)	(4.31)	(0.05)
Log PCF (total non-staple food expenditures per capita in past month)	−1.51	0.27	−0.97	−11.49***	−10.44***	1.51	0.02
	(0.99)	(1.97)	(2.62)	(1.24)	(1.81)	(1.48)	(0.02)
Log PCF * related to chief	3.46*	−2.91	4.05	1.94	−2.44	1.76	−0.03
	(2.00)	(3.66)	(4.68)	(2.35)	(3.41)	(3.22)	(0.03)

*Time-invariant baseline variables*							
Related to chief	0.63	−10.32	−0.48	9.78	16.24	−12.51	−0.20*
	(11.02)	(16.60)	(20.16)	(10.77)	(15.10)	(11.89)	(0.11)
Log(acres farmed)	6.35**	−1.27	6.37	−2.46	−5.30**	2.16	0.00
	(2.60)	(2.85)	(3.84)	(2.39)	(2.57)	(1.86)	(0.03)
Number of observations	1048	530	529	1048	530	529	530
Number of households	530	530	529	530	530	529	530
Number of villages	61	61	61	61	61	61	61
Mean of dependent variable	51.83	37.78	11.94	53.19	39.42	10.62	0.09

Note: Sample restricted to households surveyed in 2014 and asked about perceived returns to fertilizer use. Regressions for input subsidies pool years 2011 and 2012 and control for the year. 2011 input allocation information comes from 2011 survey. 2012 input and food allocations information comes from 2012 survey. Omitted age category is less than 26. Standard errors clustered at the village level. All regressions control for village fixed effects.

a Counterfactual quantities have the same distribution as actual quantities.

* Significant at 10%.

** Significant at 5%.

*** Significant at 1%.

**Table A7:** Correlations between self-reported gain and non-experimental return to fertilizer.

	(1)	(2)	(3)	(4)	(5)	(6)
	Kilograms of maize harvested			Log(kilograms of maize harvested)		
*Panel A. Levels*						
Kilograms of fertilizer used	−0.09	−0.20	−0.26			
	(0.21)	(0.36)	(0.35)			
Kilograms of improved seeds used	0.17	−0.04	−0.06			
	(0.30)	(0.50)	(0.50)			
Log(self-reported gain^a^) * kilograms of fertilizer used			1.83***			
			(0.58)			

*Panel B. Logged values*						
Log(kilograms of fertilizer used)				0.00	−5.00	−0.07
				(0.05)	(0.09)	(0.09)
Log(kilograms of improved seeds used)				0.00	0.00	0.02
				(0.03)	(0.05)	(0.05)
Log(self-reported gain^a^) * Log(kilograms of fertilizer used)						0.31*^,^**
						(0.16)
Number of observations	2502	915	915	2054	747	747
Number of households	1417	507	507	1317	480	480
Mean of dependent variable	382.40	391.80	391.80	6.45	6.47	6.47
SD of dependent variable	288.40	292.80	292.80	0.88	0.90	0.90

Note: Regressions include household fixed effects and year fixed effects. Regressions include the 2010–2011 and 2011–2012 planting seasons. All values are winsorized at the top 5%. Columns 1 and 4 include all households in the sample. The other columns are restricted to households sampled for the 2014 villager survey during which beliefs about gain from fertilizer use were elicited.

a Self-reported gain is de-meaned.

* Significant at 10%.

** Significant at 5%.

*** Significant at 1%.

## Footnotes

1 See Coady et al. (2004) for a detailed discussion of various forms of targeting.

2 See Niehaus et al. (2013) for a discussion of optimal targeting rules when programs are implemented via local, possibly corruptible, agents, but in which agents can be punished if they do not follow the rule.

3 It is not surprising that the PMT does not target on gains since the PMT focused on poverty. Generating a proxy test for potential productivity gains would be very hard in the agricultural context.

4 See Bardhan and Mookherjee (2006) for evidence on the benefits of decentralization for a credit and farming input subsidies in West Bengal.

5 Another potential mechanism to select beneficiaries is community-based targeting (CBT, where communities get together to decide on beneficiaries). Two studies that do careful comparisons between PMT and CBT in the context of cash transfer programs tend to give a modest advantage to the PMT: Alatas et al., 2013, Alatas et al., 2012 in Indonesia, where the relationship between assets and consumption is somewhat stronger than in contexts we consider; and Stoeffler et al. (2016) in Cameroon, where the CBT implementation appear poor. While the results of these two studies suggest community targeting could at best marginally improve on the chiefs' allocation, exploring the impact of community-based targeting in contexts like ours is an interesting area for future research.

6 Outside of Africa, Anderson et al. (2015) also find evidence of poor governance by elites in Maharashtra, India. Though democracy appears to be vibrant, there exists an entrenched clientelistic vote-trading system in which elite landholders are able to enact policies which lower rural wages in exchange for insurance. As in Acemoglu et al. (2014), this system is so entrenched that people report high levels of satisfaction.

7 A brief history of the coexistence of these two systems of local governance is as follows (this note relies heavily on Lihoma, 2012, Eggen, 2011, Cammack et al., 2009). Prior to colonialism, local government structures in Malawi varied across regions and ethnic groups. Most local governments included chiefs, but the role of chiefs varied between centralized systems in which the chief's authority was paramount and more decentralized, participatory systems (Lihoma, 2012). Malawi was colonized by Britain in 1891, which attempted a system of direct rule which minimized the authority of chiefs. In 1912, Britain moved towards a system of indirect rule which recognized chiefs as traditional authorities, reporting to the colonial district administrator. In 1933, traditional powers were extended such that chiefs could perform some functions of local government (such as administering communal land and arbitrating disputes in traditional courts), though chiefs were still financially dependent on colonial administrators. Beginning in 1953 and continuing until independence in 1964, the British transferred local authority from chiefs to district councils. While higher-ranked chiefs (TAs) served as ex-officio members of these councils, their powers to act unilaterally were limited (and were officially subordinate to the council itself).

8 In 2014, a village chief in Malawi received 2500 MWK (about US$6 in 2014) per month as *mswahala*, around a week's worth of labor at the prevailing casual wage.

9 Fertilizer subsidies are one of the more popular (and expensive) aid programs across the developing world, in some cases taking up significant fractions of government budgets. For example, Sri Lanka, Malawi and India spend 10–20% of their government's budget on fertilizer subsidies (Wiggins and Brooks, 2010). The countries of Zambia and Tanzania also devote 1–2% of their budget to subsidies (Baltzer and Hansen, 2011).

10 In recent years, the co-pay has increased.

11 Within each district, the District Agriculture Development Office (DADO) allocates vouchers across villages based on farming population shares (Chirwa and Dorward, 2013).

12 For example, Dorward et al. (2013) show that around 70% of households in 2013 believed the decision on voucher recipients was made by the chiefs *before* the official meeting was held.

13 We drop households that did not complete one of the three follow-up surveys (354 households, or 16.9% of the sample). We also drop households that were not administered questions about food subsidy receipt — the module to measure food subsidy receipt was introduced only partially through the endline survey, and 185 households (9.7%) were not asked these questions (9.7%). Finally, we drop 5 villages with less than 5 households in the data, since we need a sufficient number of people in each village in order to compare beneficiaries and non-beneficiaries within the same village.

14 While we do not have data these individuals in our data, in the 2016–2017 Malawi Integrated Household Survey (IHS4) households with a primary respondent who has a bank accounts have about 0.78 log points higher per capita food consumption as those in which the primary respondent does not have a bank account.

15 The reason why there are more chiefs than villages is that 19 villages were divided into multiple villages between our initial data collection in 2011–2013 and the time of the survey in 2014.

16 Given an average village size of 300 households (Table A1), the numbers imply that in an average village the chief is uncle to 16 heads of households, the brother to 11 households, the brother-in-law to 10 households, and the grandfather to 8 households. While high, these numbers are not implausible given high fertility rates.

17 The school system in Malawi is composed of 8 years of primary school and 4 years of high school.

18 Note that land allocation was not mentioned. Indeed, while customary land management traditionally falls under the chief's responsibilities, *de facto* land rarely changes hand — over the period of our panel we see no household in our sample receive or lose right to customary land.

19 Table W1 also shows summary statistics on subsidy receipt. The percentage of households receiving input subsidies increased steadily over time during this period, from 58% in 2008 to 81% in 2012. Receipt of the input subsidy is autocorrelated: 48% of households received some amount of input subsidy in all five years covered in our data, while 10% never received any input subsidy. Conditional on receiving the subsidy, the quantity of fertilizer received (summing over the two types of fertilizer, for planting and top-dressing) was about 77 and 64 kg during 2011 and 2012 respectively. This is smaller than the official package that subsidy beneficiaries are entitled to get (100 kg) due to sharing. Sharing seems to increase over time, explaining part of the growth in the coverage rate: in 2012, more households receive some subsidized inputs but they receive smaller quantities. The food subsidy of 2012 was more limited in scope than the input subsidy, reaching only 59% of households, though sharing was common for food as well. In 2012, 53% of households received both the input and food subsidy, 13.6% received neither, 5% received the food subsidy only and 27.9% received the input subsidy only.

20 These subsidies are notable for being made available to a large portion of the population, and it is possible that results would be different for subsidies that are more narrowly targeted. For example, levels of nepotism may be higher or lower in a more targeted program — we lack data to comment on this possibility.

21 The two categories excluded were recorded in the survey as “staples” and “grains/nuts.” Since the majority of households produce these items for home consumption, we expect expenditure to be negatively rather than positively correlated with total consumption for such goods: those who need to buy them from the market are those whose harvest was poor and ran out faster. Results are similar using all 12 foods (see Web Appendix).

22 We choose to compute things per capita (PC) rather than per adult equivalent (PAE) because commonly used equivalence scales between children and adults may be an underestimate of how much communities actually value children consumption (Olken, 2005). We have done the entire analysis in the paper using PAE instead of PC and the results are identical. See Deaton and Zaidi (2002) for a discussion of constructing poverty indices.

23 In principle, we could also use the January 2012 to February 2012 data since no food subsidies were distributed that year and the proceeds of the maize planted with the subsidized inputs of 2011 were not reaped until March 2012. Results look very similar using this data.

24 We use a per adult equivalent formula of 1 child under 18 equal to 0.5 adults.

25 Using a lasso regression instead of backward step-wise selection does not improve the R-squared.

26 While understanding the determinants of subsidy allocations across villages is of great interest, our data does not allow us to study this question.

27 The table also reports the probability of all or none of the villagers getting the subsidy. The proportion of villages in which everyone received the input subsidy was 9.8% in 2011 and 14.8% in 2012, while the proportion of villages in which everyone received the food subsidy in 2012 was 4.9%. In addition, nobody received the food subsidy in 4.9% of villages.

28 For the intensive margin, we construct the counterfactual PMT allocation keeping the distribution of input subsidy values the same as under the chief, but assigning the largest value subsidy to the household with the lowest PMT score, the second largest value to the household with the second lowest PMT score, etc. This inflates the targeting performance of the PMT compared to allocating fixed subsidy values to every eligible household. This is the relevant benchmark insofar as there is no reason (other than logistical constraints) why subsidy amounts under the PMT cannot be varying with the PMT score.

29 We use the regression-adjusted point estimate (12 percentage points) and the overall likelihood of getting the subsidy among non-kin (31.8%).

30 A concern may be that food expenditure is not a good measure of need. There is undoubtedly measurement error in food expenditure, which would tend to flatten the gradient in PCF for both the chiefs and the PMT. In addition, our measure is based on expenditure, not consumption, and consumption will depart from expenditure if people receive transfers. To address this, in the Web Appendix we perform robustness checks with a variety of other measures of need, including total (measured) expenditures, total food expenditures on all categories, the share of food in expenditures, the percent of days in which respondents report not having enough food, and an indicator equal to 1 if a respondent reported being worried about food. All measures are strongly correlated, e.g. the (within) R-squared for total and food expenditure is 0.37–0.39. When we redo our main analysis using total expenditure and total food expenditure instead of non-staple food expenditure, results look qualitatively similar, though error rates for chiefs are higher with these measures than for non-staple food (Web Appendix Table W3). Our interpretation of the difference between these results and the previous set is that chiefs are able to target people who are poor, even conditional on total expenditures. Indeed, our expenditures module did not include all possible expenditures categories. To the extent that Engel curves are not linear (see Ligon, 2017 for evidence that they are not among rural households in Uganda), and the categories we did not measure represent a higher share of total expenditures for relatively richer households, our measure of total expenditures may be less informative about household's relative neediness than expenditures on an exhaustively measured list of items in a subcategory (e.g. food). We also redo our analysis of the correlates of subsidy receipt in Web Appendix Table W4, using these alternative measures of expenditures. Results are unchanged: we still find strong evidence that chief's kin are favored.

31 Another concern might be that the food and input subsidies were decided jointly, rather than sequentially. This is not the case here, however — the food subsidy was not expected and was announced after the input subsidy had been delivered.

32 The main issue here is that farmers may have bought some fertilizer even in the absence of the subsidy, and so some of the subsidized input may be inframarginal. Another potential issue would be that returns might be concave in quantities. We argue this is unlikely in our setting, since the size of the subsidy does not cover the whole farm. Farmers typically use a given amount of fertilizer per plant, so that an increase in inputs would involve an increase in acreage under fertilizer rather than an increase in fertilizer per plant. While it still may be the case that fertilizer is allocated to parcels of land with higher returns first, we argue that average and marginal returns are likely correlated. Nevertheless, we acknowledge that our measure is not perfect.

33 The analysis in Table 4 only controls for log farm size, log PCF, and relation to chief: we omit other household controls such as demographics, since these controls themselves are predictors of log gains as shown in Table A5. Including all other controls attenuates coefficients (see Web Appendix Table W5). The gap in coefficients on log gains between the chief and counterfactual PMT allocation remains large, however, and for our primary outcome — the value gap between the fertilizer and food subsidies, the object of the model's prediction — the coefficient on log gains remains large and significant at 5% for the chief's allocation and small and insignificant under the PMT. The results are also robust to controlling semi-parametrically for farm size instead of using a log-linear specification (Panel A of Web Appendix Table W6). Panel B of Table W6 uses *per acre yield gain* as the independent variable of interest. The results are weaker than for total yield gain, which we argue is not surprising since the per acre yields are not as important as total gains (since on small enough plots of land, high returns do not translate into large effects on total income). Nevertheless, the coefficient is of the correct sign. Note that we cannot regress input per acre on yield gain per acre because this would generate division bias and hence a spurious, very significant relationship between the two.

34 As above, another question is whether chiefs have better information on relatives. We check this in Table A6 in which we include interactions between productivity and kinship. We find little evidence of better targeting among relatives.

35 We are extremely grateful to Santiago Saavedra for obtaining the administrative records and performing the matching analysis and verification surveys.
