# Supplementary material for: Decentralization and efficiency of subsidy targeting: Evidence from chiefs in rural Malawi
Source: J Public Econ. 2020 May;185:104047. doi: 10.1016/j.jpubeco.2019.07.006 (PMC7224518; doi:10.1016/j.jpubeco.2019.07.006)
Supplement: Supplementary file 1 — Supplementary material. [file mmc1.pdf]

Decentralization and efficiency of subsidy targeting: Evidence from chiefs in rural Malawi

Basurto, Dupas and Robinson

Journal of Public Economics

Web Appendix
(Not for Publication)

Figure W. Distributions of key variables of interest

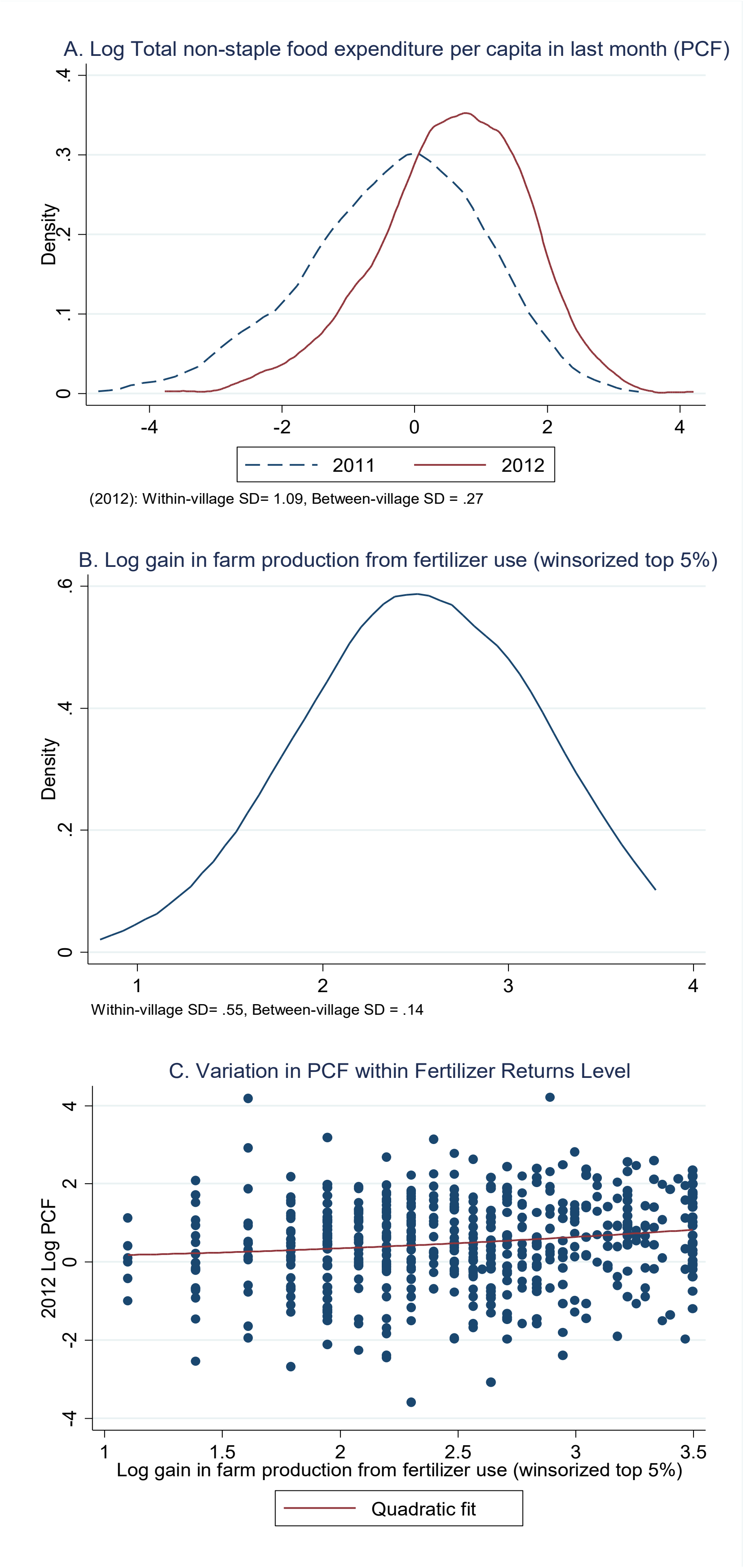

Notes: Gain in farm production expressed in 50 Kg bag units.

Table W1. Exposure to Subsidy Programs

	(1) Mean	(2) Std. Dev.
Panel A. Input subsidy		
Received input subsidy in 2008	0.58	
....in 2009	0.66	
....in 2010	0.73	
....in 2011	0.77	
....in 2012	0.80	
If any, kgs of fertilizer received in 2011	81.58	26.54
.... 2012	64.22	25.47
If any, kgs of seeds received in 2011	5.07	3.44
.... 2012	4.80	3.05
If received subsidy, value of 2011 package[1]	72.28	24.14
....2012 package	58.07	22.68
Received input subsidy all 5 years	0.47	
Never received input subsidy	0.10	
Sharing (based on 2014 villagers survey, N=504)		
Received voucher and didn't share	0.14	
Received voucher and shared	0.46	
Received share of someone's voucher	0.30	
Didn't receive a voucher or share	0.10	
Who decided the voucher would be shared? (Asked of voucher recipients)		
Village Chief	0.85	
Villagers themselves	0.13	
Other	0.02	
Who decided with whom the voucher would be shared? (Asked of voucher recipients)		
Village Chief	0.73	
Villagers themselves	0.23	
Other	0.04	
Who decided with whom the voucher would be shared? (Asked of share recipients)		
Village Chief	0.85	
Villagers themselves	0.09	
Other	0.06	
Panel B. Food Subsidy		
Received food subsidy in 2012	0.59	
If received subsidy, value of package	72.00	37.40
Received both food and input subsidy in 2012	0.53	
Sharing (based on 2014 villagers survey, N=504)		
Who decided with whom the food would be shared?		
Village Chief	0.75	
Group Village Chief	0.03	
Villagers themselves	0.13	
Other	0.09	

Note: All monetary amounts are in US dollars. Exchange rate was roughly 150 MWK to $1 at the time of the baseline, and it was 300 MWK to $1 in late 2012.

Table W2. Predictive power of PMT formula across datasets

	(1)	(2)	(3)	(4)
Dependent variable: log PCF	Malawi IHS3, BDR variables only	Malawi, All IHS3 Variables	Kenya	Uganda
Household size (divided by 10)	-5.82***	-5.63***	-3.43***	-3.09***
	(0.66)	(0.68)	(0.42)	(0.35)
Household size (divided by 10) squared	2.66***	2.58***	1.12***	1.10***
	(0.56)	(0.57)	(0.26)	(0.28)
Number of children under 5 (divided by 10)	-1.24***	-1.16***	-	-
	(0.39)	(0.42)		
Total number of children (divided by 10)	1.01**	0.84*	-	-
	(0.42)	(0.46)		
Log durable assets	0.17***	0.17***	0.19***	0.28***
	(0.03)	(0.03)	(0.04)	(0.03)
Log animal assets	-	-	-	0.05***
				(0.02)
Owns land	-	-0.18*	-	-
		(0.09)		
Owns land * log acres owned	-	-	-	-
Widowed or Divorced Female Head	-0.26***	-0.24***	-0.83***	-0.20***
	(0.09)	(0.09)	(0.09)	(0.06)
Age of respondent (divided by 100)	-	-	5.87***	-0.89***
			(1.41)	(0.21)
Age of respondent (divided by 100) squared	-0.38**	-0.40**	-6.76***	-
	(0.18)	(0.18)	(1.46)	
Highest education within household	0.03**	0.03**	0.03**	-
	(0.01)	(0.01)	(0.01)	
Household head is literate	-	-	-	0.12**
				(0.05)
Home has mud or dirt floors	-	-	-	-0.17***
				(0.05)
Home has thatch roof	-0.37***	-0.35***	-	-
	(0.08)	(0.08)		
Home has mud or dirt walls	-	-	-0.61***	-0.11*
			(0.23)	(0.07)
Toilet is private covered latrine	-0.26**	-	-	-
	(0.11)			
Toilet is uncovered latrine	-0.28**	-	-	-
	(0.12)			
No toilet	-0.30*	-	-	-
	(0.16)			
Water source is public tap	0.48***	0.48***	-	-
	(0.12)	(0.13)		
Water source is well	-	-	-	-
Water source is piped water	-	-	-	-
Has electricity	0.35*	0.39**	-	-
	(0.18)	(0.18)		
Has a mobile phone	-	-	-	-
Main occupation = vendor	-	-		-0.26***
				(0.05)
Main occupation = owner of other business	0.29***	0.30***	-	0.43***
	(0.09)	(0.09)		(0.08)
Variables in IHS3 but not BDR				
Value of house (USD)	-	-	-	-
Has trash pit for garbage	-	-	-	-
R-squared	0.40	0.39	0.31	0.28
Households	763	763	845	2160
Villages	48	48		

Notes: Columns 1 and 2: Data comes from Malawi Integrated Household Survey Wave 3 (IHS3). Columns 3 and 4: Data from surveys conducted in 2010 in Kenya (Dupas, Keats and Robinson 2016) and Uganda (Dupas et al. 2016). Dependent variable is total household log per capita food expenditures (monhtly equivalaent), in USD. Sequential selection of variables done using Stata backward stepwise regression. Standard errors, clustered by village, in parentheses. *, **, and *** denote significance at 10%, 5%, and 1%.

Table W3. Targeting errors, based on total expenditures and total food expenditures

	(1)	(2)	(3)	(4)	(5)	(6)
Neediness proxied using:	Total expenditures			Total food expenditures		
	2011 Input Subsidy	2012 Input Subsidy	2012 Food Subsidy	2011 Input Subsidy	2012 Input Subsidy	2012 Food Subsidy
Simple error rate under following allocation mechanism:[1]						
Chief (True allocation)	0.155	0.145	0.169	0.153	0.145	0.165
PMT (Counterfactual)	0.135	0.116	0.140	0.116	0.102	0.123
PMT (Counterfactual) based on IHS3 formula	0.138	0.120	0.143	0.127	0.099	0.137
Random (Counterfactual)	0.152	0.126	0.162	0.153	0.125	0.162
P-val Chiefs = PMT	0.075	0.006	0.003	0.005	<.001	<.001
P-val Chiefs = PMT (IHS3)	0.080	0.022	0.014	0.025	<.001	0.003
P-val Chiefs = Random	0.754	0.100	0.485	0.992	0.061	0.758
P-val PMT = Random	<.001	<.001	<.001	<.001	<.001	<.001
Mean squared error in log consumption under following allocation mechanism:[2]						
Chief (True allocation)	0.651	0.512	0.796	0.487	0.542	0.881
PMT (Counterfactual)	0.350	0.236	0.495	0.281	0.288	0.618
PMT (Counterfactual) based on IHS3 formula	0.499	0.329	0.616	0.422	0.360	0.866
Random (Counterfactual)	0.699	0.826	2.081	0.524	1.002	2.559
P-val Chiefs = PMT	0.036	0.010	0.032	0.463	0.016	0.822
P-val Chiefs = PMT (IHS3)	0.767	<.001	<.001	0.611	<.001	<.001
P-val Chiefs = Random	0.009	<.001	<.001	<.001	<.001	<.001
P-val PMT = Random	<.001	<.001	<.001	<.001	<.001	<.001

Notes: See Table 2 notes

Table W4. Multivariate regressions with total expenditures or total food expenditures

	(1)	(2)	(3)	(4)	(5)	(6)	(7)	(8)
	Actual (Chief's) allocations				Counterfactual PMT allocation			
	Got input subsidy	Value (USD)	Got food subsidy	Value (USD)	Eligible for input subsidy	Value (USD)[a]	Eligible for food subsidy	Value (USD)[a]
Panel A. Total expenditures								
Log per capita total expenditures	-0.01*	0.06	-0.04***	-3.06**	-0.05***	-5.32***	-0.03***	-3.54***
	(0.01)	(0.69)	(0.01)	(1.24)	(0.01)	(0.45)	(0.01)	(0.63)
Related to chief	0.04	3.19*	0.12***	10.99***	-0.01	1.93	0.04	3.07*
	(0.02)	(1.71)	(0.03)	(2.85)	(0.02)	(1.17)	(0.03)	(1.79)
Number of Observations	3094	3043	1559	1559	3118	3043	1559	1559
Number of Households	1558	1558	1559	1559	1559	1558	1559	1559
Number of Villages	61	61	61	61	61	61	61	61
Mean of dependent variable	0.78	50.47	0.59	42.03	0.78	50.47	0.59	42.03
Panel B. Total food expenditures								
Log per capita total food expenditures	-0.01*	-0.39	-0.04***	-2.85**	-0.03***	-3.46***	-0.02**	-1.87***
	(0.01)	(0.58)	(0.01)	(1.22)	(0.01)	(0.41)	(0.01)	(0.65)
Related to chief	0.04	3.14*	0.11***	10.85***	-0.01	1.8	0.04	3.15*
	(0.02)	(1.71)	(0.03)	(2.86)	(0.02)	(1.20)	(0.03)	(1.80)
Number of Observations	3094	3043	1559	1559	3118	3043	1559	1559
Number of Households	1558	1558	1559	1559	1559	1558	1559	1559
Number of Villages	61	61	61	61	61	61	61	61
Mean of dependent variable	0.78	50.47	0.59	42.03	0.78	50.47	0.59	42.03

Note: Regressions control for all variables in Table 3, but only key variables are shown. Standard errors clustered at the village level. All regressions include village fixed effects.

[a] Counterfactual quantities have the same distribution as actual quantities.

* significant at 10%; ** significant at 5%; *** significant at 1%

Table W5. Productive efficiency with longer list of controls

	(1)	(2)	(3)	(4)	(5)	(6)	(7)
	Actual (Chief's) allocations			Counterfactual PMT allocation			
	Value (USD) of input subsidy	Value (USD) of food subsidy	Value gap (input-food)	Value (USD) of input subsidy under PMT[a]	Value (USD) of food subsidy under PMT[a]	Value gap (input-food) under PMT	Ever Lobbied Chief to try to get Input Subsidy
Log (gain in farm production from fertilizer use)	2.58	-0.45	7.29**	-2.53	-4.96*	2.80	0.00
	(1.58)	(2.54)	(3.37)	(1.88)	(2.92)	(2.92)	(0.03)
Log (total non-staple food expenditures per capita in past month)	1.01	1.83	-1.29	-5.69***	-4.38***	0.05	0.00
	(0.87)	(1.93)	(2.57)	(1.06)	(1.48)	(1.59)	(0.01)
Time-Invariant Baseline Variables							
Related to chief	0.89	7.55*	-4.73	4.70**	5.28*	-0.30	0.04
	(3.06)	(3.97)	(5.38)	(2.30)	(2.93)	(3.55)	(0.03)
Log (acres farmed)	3.21	-4.38	7.09*	-5.66**	-9.49***	4.71**	0.01
	(2.67)	(2.95)	(3.77)	(2.46)	(2.23)	(1.81)	(0.03)
Years of education (divided by 10)	1.83	-8.01	10.35	-30.05***	-33.16***	7.90	0.01
	(5.57)	(7.21)	(8.21)	(4.22)	(5.05)	(4.96)	(0.06)
Widowed or divorced female	-1.35	0.80	-1.93	10.17***	16.49***	-4.99	0.00
	(2.76)	(3.93)	(4.40)	(2.55)	(3.65)	(3.78)	(0.03)
Household size (divided by 10)	-0.22	3.68	-0.10	50.23***	69.14***	-22.05*	0.02
	(6.19)	(9.64)	(12.86)	(9.00)	(13.82)	(11.87)	(0.08)
Respondent age: 2nd quartile (26-35)	11.32**	4.23	4.37	1.52	-1.37	2.75	-0.10**
	(4.32)	(5.82)	(6.95)	(3.52)	(4.75)	(4.55)	(0.05)
Respondent age: 3rd quartile (36-51)	15.68***	11.85	1.46	4.42	-1.21	4.44	-0.12*
	(4.67)	(7.53)	(7.83)	(3.70)	(4.67)	(5.30)	(0.06)
Respondent age: highest quartile (over 52)	19.07***	24.02***	-8.67	13.84***	12.83**	-0.52	-0.13**
	(4.93)	(6.95)	(7.24)	(4.62)	(5.77)	(6.16)	(0.06)
Log (value of animals owned)	0.62	0.37	0.08	-2.88***	-3.22**	0.27	0.03
	(1.09)	(1.46)	(1.79)	(0.99)	(1.43)	(1.41)	(0.02)
Shocks							
Experienced drought or flood (past 3 months)	0.33	4.34	-3.60	-2.56	0.62	0.05	-0.08**
	(3.65)	(4.81)	(6.17)	(3.05)	(4.69)	(4.84)	(0.03)
Experienced cattle death or crop disease (past 3 months)	3.47	-1.42	5.25	2.23	5.02	-3.24	-0.02
	(2.54)	(4.29)	(4.49)	(2.30)	(3.11)	(3.12)	(0.02)
Information from villager survey							
Ever made a payment to the village chief	-4.33*	-7.70*	5.38	-3.95	-3.56	0.28	0.01
	(2.23)	(4.39)	(4.79)	(2.38)	(3.63)	(3.51)	(0.03)
Number of Observations	1048	530	529	1048	530	529	530
Number of Households	530	530	529	530	530	529	530
Number of Villages	61	61	61	61	61	61	61
Mean of dependent variable	51.83	37.78	11.94	52.96	40.53	9.26	0.09

Note: Sample restricted to households surveyed in 2014 and asked about perceived returns to fertilizer use. Regressions for input subsidies pool years 2011 and 2012 and control for the year. 2011 input allocation information comes from 2011 survey. 2012 input and food allocations information comes from 2012 survey. Omitted age category is less than 26. Standard errors clustered at the village level. All regressions control for village fixed effects.

* significant at 10%; ** significant at 5%; *** significant at 1%

Table W6. Productive efficiency results: alternative specifications

	(1)	(2)	(3)	(4)	(5)	(6)	(7)
	Actual (Chief's) allocations			Counterfactual PMT allocation			Ever Lobbied Chief to try to get Input Subsidy
Panel A. Non-parametric controls for farm size	Value (USD) of input subsidy	Value (USD) of food subsidy	Value gap (input-food)	Value (USD) of input subsidy under PMT[a]	Value (USD) of food subsidy under PMT[a]	Value gap (input-food) under PMT	
Log (gain in farm production from fertilizer use)	4.34**	-0.01	8.46**	-2.69	-4.65	2.79	0.00
	(1.72)	(2.91)	(3.46)	(2.54)	(3.19)	(2.79)	(0.03)
Log (total non-staple food expenditures per capita in past month)	-0.05	-0.66	0.34	-11.60***	-10.90***	1.87	0.02
	(0.75)	(1.56)	(2.16)	(1.04)	(1.95)	(1.71)	(0.01)
Time-Invariant Baseline Variables							
Related to chief	2.36	11.01***	-7.67	7.51***	7.92*	-0.93	0.03
	(2.93)	(3.97)	(5.28)	(2.52)	(4.24)	(3.91)	(0.03)
Q2 of acres farmed	4.80	6.58	-4.62	0.26	-8.76	5.28	-0.02
	(4.20)	(6.97)	(7.40)	(4.41)	(6.23)	(4.80)	(0.04)
Q3 of acres farmed	9.37***	5.22	1.20	1.36	-2.94	1.44	-0.01
	(3.35)	(4.78)	(6.00)	(2.84)	(4.47)	(3.66)	(0.04)
Q4 of acres farmed	7.61*	-1.41	9.47	-4.75	-10.37**	4.99	-0.02
	(4.02)	(4.53)	(5.81)	(3.73)	(4.74)	(3.08)	(0.04)
Panel B. Targeting on productivity per acre							
Log (per acre yield gain from fertilizer use)	2.63	1.37	5.96*	-1.50	-1.98	0.96	-0.01
	(1.88)	(3.15)	(3.52)	(2.52)	(3.18)	(2.56)	(0.03)
Log (total non-staple food expenditures per capita in past month)	0.01	-0.74	0.56	-11.70***	-11.10***	2.02	0.01
	(0.77)	(1.56)	(2.16)	(1.03)	(1.94)	(1.73)	(0.01)
Time-Invariant Baseline Variables							
Related to chief	2.11	10.96***	-7.99	7.38***	7.90*	-0.95	0.03
	(2.94)	(3.99)	(5.23)	(2.54)	(4.28)	(3.94)	(0.03)
Q2 of acres farmed	6.20	7.30	-1.44	-0.78	-9.91	5.80	-0.02
	(4.48)	(7.24)	(7.43)	(4.74)	(6.45)	(4.94)	(0.04)
Q3 of acres farmed	11.97***	6.26	6.70	-0.22	-4.90	2.40	-0.02
	(3.91)	(5.54)	(6.52)	(3.78)	(5.22)	(3.91)	(0.04)
Q4 of acres farmed	11.92**	0.33	18.82***	-7.45	-13.86**	6.74	-0.03
	(4.79)	(5.69)	(6.86)	(4.85)	(6.27)	(4.50)	(0.04)
Number of Observations	1038	525	524	1038	525	524	525
Number of Households	525	525	524	525	525	524	525
Number of Villages	61	61	61	61	61	61	61
Mean of dependent variable	52.20	38.05	11.98	53.39	40.86	9.41	0.09

Note: Sample restricted to households surveyed in 2014 and asked about perceived returns to fertilizer use. Regressions for input subsidies pool years 2011 and 2012 and control for the year. 2011 input allocation information comes from 2011 survey. 2012 input and food allocations information comes from 2012 survey. Omitted age category is less than 26. Standard errors clustered at the village level. All regressions control for village fixed effects.

* significant at 10%; ** significant at 5%; *** significant at 1%

Table W7. Perceived Within-Village Heterogeneity among Village Chiefs

Can you easily categorize households in the village with land better suited for fertilizer and those with land not so well suited for fertilizer?	
Yes	0.86
Can you easily categorize households in the village in two groups, those who are very poor and those who are less poor?	
Yes	0.96
Do you know which families in the village are having specific difficulty with money at a given time?	
I know how everyone is doing	0.65
I know how some people are doing	0.32
I do not know	0.04
Do you know who is likely to have money to buy fertilizer for the coming planting season and who will not?	
I know how everyone is doing	0.49
I know how some people are doing	0.27
I do not know	0.24
Number of observations	79

Notes: From survey of village chiefs conducted in 2014. See text for details.
